# Bacterial Toxins Are a Never-Ending Source of Surprises: From Natural Born Killers to Negotiators

**DOI:** 10.3390/toxins13060426

**Published:** 2021-06-17

**Authors:** Maria Lopez Chiloeches, Anna Bergonzini, Teresa Frisan

**Affiliations:** Department of Molecular Biology and Umeå Centre for Microbial Research (UCMR), Umeå University, 901 87 Umeå, Sweden; maria.chiloeches@umu.se (M.L.C.); anna.bergonzini@umu.se (A.B.)

**Keywords:** bacterial genotoxins, pore-forming toxins, immunoregulation, cytokines, polarization immune response, innate and adaptive immunity

## Abstract

The idea that bacterial toxins are not only killers but also execute more sophisticated roles during bacteria–host interactions by acting as negotiators has been highlighted in the past decades. Depending on the toxin, its cellular target and mode of action, the final regulatory outcome can be different. In this review, we have focused on two families of bacterial toxins: genotoxins and pore-forming toxins, which have different modes of action but share the ability to modulate the host’s immune responses, independently of their capacity to directly kill immune cells. We have addressed their immuno-suppressive effects with the perspective that these may help bacteria to avoid clearance by the host’s immune response and, concomitantly, limit detrimental immunopathology. These are optimal conditions for the establishment of a persistent infection, eventually promoting asymptomatic carriers. This immunomodulatory effect can be achieved with different strategies such as suppression of pro-inflammatory cytokines, re-polarization of the immune response from a pro-inflammatory to a tolerogenic state, and bacterial fitness modulation to favour tissue colonization while preventing bacteraemia. An imbalance in each of those effects can lead to disease due to either uncontrolled bacterial proliferation/invasion, immunopathology, or both.

## 1. Introduction

**Figure 1:**
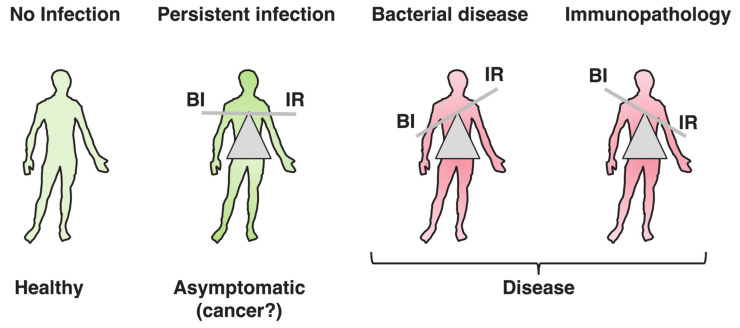
Toxins’ contributions to persistent or acute pathological bacterial conditions. Bacterial genotoxins (BTGXs) and pore-forming toxins (PFTs) can contribute to the modulation of the host’s immune response (IR) during bacterial infection (BI). When BTGXs and PFTs contribute to avoid complete bacterial clearance by the IR and limit immunopathology, the infection can persist for long time, eventually asymptomatically (where chronic carrier status can be associated with pathologies such as cancer). However, when this delicate equilibrium is broken, due to high toxin- and bacterial-induced tissue damage or uncontrolled immune responses, pathological conditions develop.

The word toxin according to the definition by Merriam-Webster is “a poisonous substance that is a specific product of the metabolic activities of a living organism and is usually very unstable, notably toxic when introduced into tissues, and typically capable of inducing antibody formation,” which entails the concept of tissue destruction (https://www.merriam-webster.com/dictionary/toxin#note-1). Results from the past decades have highlighted the possibility that the biological function of bacterial toxins, key virulence factors in bacterial pathogenesis [1,2], may not exclusively be to cause damage to the host tissue, allowing bacteria to colonize, invade, and spread. Bacterial toxins also have a more subtle ability to tweak the host’s immune response towards a delicate balance, which prevents tissue damage and immunopathology. Concomitantly, this may limit pathogen clearance, allowing the establishment of a niche for successful colonization, which may lead to persistent infections [3]. When this delicate equilibrium is broken, due to high toxin (bacteria)-induced tissue damage or uncontrolled immune responses, pathological conditions develop, as summarized in Figure 1. The killer vs. negotiator paradox is directly connected to another highly relevant issue in bacterial pathogenesis, namely the amount of toxin that is produced during the course of an in vivo infection. The local toxin concentration may vary from toxic amounts (the killer) to subtoxic doses (the negotiator) and can be influenced by many parameters, such as the duration of infection, the infection dose, and the host/microbiota microenvironment.

**Figure 2:**
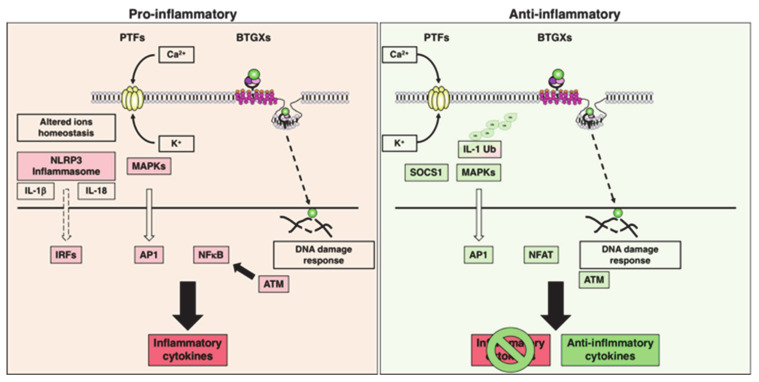
Bacterial genotoxins (BTGXs) and pore-forming toxins (PFTs) have different modes of action on the target cells but share common pathways and outcomes as immunomodulators resulting in either: (i) secretion of pro-inflammatory cytokines (key regulatory effectors described in the text are highlighted in red; left panel); (ii) suppression/degradation of pro-inflammatory cytokines and/or secretion of anti-inflammatory mediators (key regulatory effectors described in the text are highlighted in green; right panel). AP1: Activator Protein 1 (transcription factor); ATM: ataxia telangiectasia mutated (kinase); IRFs: interferon-responsive factors (transcription factors); MAPKs: mitogen-activated protein kinases; NFAT: nuclear factor of activated T cells (transcription factor); NFκB: nuclear factor-kB (transcription factor); SOCS1: suppressor of cytokine signalling 1; Ub: ubiquitin.

**Figure 3:**
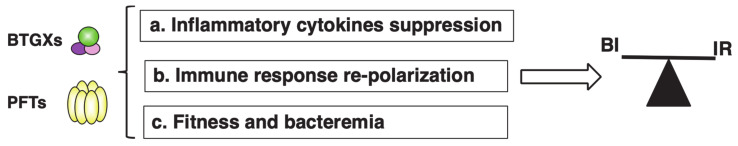
Different immunosuppressive effects of BTGXs and PFTs. BTGXs and PFTS can modulate the host’s immune response by several non-mutually exclusive mechanisms: (a) suppression of pro-inflammatory cytokine production/secretion (PFT); (b) re-polarization of the host’s immune response from a pro-inflammatory (Th1 and Th17) to a tolerogenic and tissue repair-prone response (Th2); and (c) toxin expression can reduce the fitness of the bacterium to survive hostile environments such as the blood, favouring tissue colonization and preventing bacteraemia. This can lead to a balance between bacterial infection/colonization (BI) and uncontrolled activation of the immune response (IR).

In this review, we will focus on the immunomodulatory role of two families of bacterial toxins: (a) genotoxins (BTGXs) and (b) pore-forming toxins (PFTs). We have chosen these two effectors, among all the bacterial exotoxins, which may have similar outcomes on the regulation of the host response because they have very different modes of action and target the two innermost and outermost essential structures of a cell, respectively: the DNA and the plasma membrane; the alpha and omega. BTGXs damage the host DNA [4] and PFTs compromise the integrity of the host membranes [5] (Table 1 and Table 2 and Figure 2). However, as immunomodulators, they can achieve very similar outcomes, often targeting common node regulators such as transcription factors (e.g., NFκB) and protein kinases, highlighting the relevance of their activities during host–pathogen interactions (Figure 3). For both toxin families, the best-characterised immunomodulatory effect is promotion of proinflammatory responses [2,4].

BTGXs induce DNA damage in target host cells [6]. The subsequent cellular response is characterised by secretion of a wide variety of pro-inflammatory cytokines (IL-1β, IL-6, IL-8, and TNFα) within a period of 5 to 72 h post-intoxication in vitro [4], possibly via NFκB activation induced by the cellular DNA damage response (DDR) pathway [7]. However, there is also a late pro-inflammatory effect (from 4 days post-intoxication onwards), associated with induction of senescence, a permanent quiescent status that prevents growth of DNA-damaged cells. Senescent cells secrete a highly pro-inflammatory secretome, known as senescence-associated secretory phenotype (SASP) [8,9].

The pro-inflammatory response mediated by PFTs is linked to their capacity to: (i) induce necrosis or pyroptosis, and the subsequent release of damage-associated molecular pattern (DAMPs) into the extracellular environment [5,10]; (ii) activation of the NLR family pyrin domain-containing 3 (NALP3) inflammasome through diverse mechanisms such as alteration of the ion homeostasis (efflux of potassium K^+^ and influx of calcium Ca^2+^), or rupture of the phagosome/lysosome compartments, resulting in release of the pro-inflammatory cytokines IL1β and IL-18 [11]; (iii) activation of the transcription factor NFκB via induction of Ca^2+^ oscillation at sublethal doses, leading to transcriptional activation and secretion of a broad panel of pro-inflammatory cytokines, such as IL-6 and IL-8 [12,13].

However, another interesting aspect of the immunomodulatory effects of PFTs and BTGXs is their anti-inflammatory activity, which goes beyond their capacity to induce cell death in general or specifically in cells of the innate or adaptive immune system [4,14,15]. This outcome relies on the manipulation of the immune response, resulting in: (i) suppression of pro-inflammatory cytokines, (ii) re-polarization of the immune response, and (iii) balance between toxin production and bacterial fitness (Figure 3). Thus, these effectors can take control and modulate the mechanisms of host tolerance to infection, defined as “reduction of the negative impact of infection on host fitness without affecting the pathogen burden” [16] to their own advantage. The immunosuppressive role of BTGXs and PFTs will be the main topic of this review.

## 2. Bacterial Genotoxins

### 2.1. General Overview and Mode of Action

Pathogenic bacteria are masters in manipulating the biological processes of infected eukaryotic cells and have the capacity to exploit them for their own advantage. Their main goal is to invade and replicate within the host, possibly in a stealth manner. To achieve the latter, pathogens have developed a variety of weapons during their evolutionary process. Nowadays, one of the most unconventional weapons that bacteria possess is represented by the family of genotoxins.

Bacterial genotoxins are effectors widely diffused among Gram-negative bacteria, which exert their cytotoxic effect by acting as a Type I deoxyribonuclease (DNase I), inducing DNA single-strand breaks (SSBs) and DNA double-strand breaks (DSBs), or as DNA inter-strand cross-linkers. The DNA damaging activity causes cytoplasmic and nuclear distension in targeted cells, eventually leading to cell cycle arrest, apoptosis, or senescence. Three families of genotoxins have been described up to date: the cytolethal distending toxins, the typhoid toxin, and colibactin.

#### 2.1.1. Cytolethal Distending Toxins

The cytolethal distending toxins (CDTs) include a family of bacterial toxins produced by a variety of pathogenic bacteria, such as *Escherichia coli, Aggregatibacter actinomycetemcomitans, Haemophilus ducreyi, Shigella dysenteriae, Campylobacter* species, and *Helicobacter* species. In order to simplify the specification of CDTs, we use the nomenclature proposed by Cortes-Bratti, where each CDT is referred to by designating the initials of the producing bacterium followed by CDT (e.g., *Haemophilus ducreyi* CDT: HdCDT) [17,18].

CDTs are AB_2_ trimers constituted by the enzymatically active subunit A (CdtB), which is capable of cleaving the DNA of host cells, and B subunits (CdtA and CdtC), which represent the binding components. The entire complex is encoded from a single operon and all three gene products, CdtA, CdtB, and CdtC, are required to confer full activity to the holotoxin.

CdtA and CdtC are lectin-type molecules and represent the binding moiety to the cell surface, an essential mechanism for the subsequent delivery of the active subunit to intracellular compartments. The crystal structure of the CdtB subunit shows the canonical four-layered fold for DNase-I families of enzymes, consisting of a central 12-stranded β-sandwich packed between outer α-helices and loops on each side of the sandwich. In common with DNase I, CdtB possesses two conserved histidine residues (HdCdtB His 160 and His 274), which are critical for toxin activity [19].

The capacity of CDTs to induce cell cycle arrest has been extensively demonstrated in vitro by induction of CDT-mediated fragmentation of purified plasmid [20,21] or DNA fragmentation upon intoxication of mammalian cells [22]. As a consequence of DNA damage, cells activate the DNA damage response, leading to cell cycle arrest [6,23,24].

The mechanisms by which CdtB is internalized and exerts its cytotoxic activity are only partially understood and might differ from CDT to CDT. Several pieces of evidence indicate that membrane cholesterol represents an essential ligand for CDT, which is necessary for the internalization of CdtB and cellular intoxication. This has been proven experimentally by cholesterol depletion from the plasma membrane with methyl-β-cyclodextrin-disrupting lipid rafts, which subsequently abrogated HdCDT, *Actinobacillus actinomycetemcomitans* CDT (AaCDT), and *Campilobacter jejuni* CDT (CjCDT) binding and intoxication [25,26,27]. Conversely, the *E. coli* CDT (EcCDT) has been reported to bind N-linked fucose-containing complex carbohydrates on cell surfaces [28].

Genes coding for endosomal proteins have been shown to be essential for the cytotoxic activity of CDTs, and thus, it is conceivable that, upon internalization, trafficking via the endosomal compartment is a common feature for many CDTs [29], with the exception of EcCDT, which has been shown to bypass the transit through the late endosome [30].

From the endosomal compartment, AaCDT and HdCDT are retrogradely transported to the Golgi complex and subsequently to the endoplasmic reticulum (ER) [25]. The retrograde transport via the Golgi complex is further supported by Battaglia et al., who demonstrated that AaCdtB translocation is dependent on synaptogyrin-2 and disrupted by Retro-2, an inhibitor of the endosome-Golgi transport [31]. Another key protein that regulates endocytosis and endosomal trafficking is Rab5a, which has been shown to play a role in mediating CdtB-induced inflammatory response and cytotoxicity [32].

The ER to cytosolic translocation of the CdtB subunit may be dependent on the ER-associated degradation (ERAD) pathway [33]. However, the translocation from the ER to the nuclear compartment is still not clear and may differ among several members of the CDT family, as previously suggested [29].

#### 2.1.2. Typhoid Toxin

The genes of the typhoid toxin (TT) have been identified in *S. enterica* serovar Typhi, Paratyphi, Schwarzengrund, 9,12:l,v:-, and Bredeney and in *S. enterica* subspecies *arizonae* and *diarizonae*, and *javiana* [34,35,36,37,38]. Different from CDTs, TT possesses an A_2_B_5_ structure, which includes two enzymatically active subunits and a pentameric ring that is responsible for binding the host cell, allowing the subsequent internalization of the toxin. The active moieties are composed of the CdtB and PltA subunits, which have minimal interaction and are linked by a disulphide bond between Cys214 of PltA and Cys269 of CdtB. Crystallographic studies revealed that PltA exhibits a structure similar to the pertussis-like toxin, with ADP ribosyl transferase activity, whose cellular targets have not been identified yet. CdtB shows a similar structure to the CdtB subunit present in the CDT holotoxin [35]. The binding component is constituted by the pentameric PltB ring, whose five monomers represent the base of the reverse pyramidal structure of the complex [35] and favour the binding to the surface glycoproteins sialoglycans, with an acetyl neuraminic acid as terminal domain, preferentially expressed by human cells [39].

After its receptor-mediated uptake, similar to CDT, TT is retrogradely transported to the ER. In this compartment, the toxin is disassembled by reduction of the disulphide bond between PltA and CdtB, and both components can be individually translocated to the cell cytosol [40].

The typhoid toxin is expressed once the bacterium has been internalized by a host cell, where it is secreted into the lumen of a *Salmonella*-containing vacuole, packaged in bacterial outer membrane vesicles (OMVs) that can be secreted via anterograde transport towards the cellular cortex on the microtubule and actin tracks. Upon release into the extracellular environment, the TT-loaded OMVs can be internalized by bystander cells in a dynamin-dependent manner. Induction of DNA damage further requires retrograde transport via the Golgi complex [41].

#### 2.1.3. Colibactin

The genotoxin colibactin is a metabolite produced by the polyketide synthase (*pks*) island harboured by extraintestinal pathogenic *E. coli* (ExPEC) and other members of *Enterobacteriaceae.* The *pks* island is present in pathogenic, commensal, and even probiotic bacterial strains [24]. Large-scale whole-genome-based studies have investigated the *pks* island phylogeny and suggested the possibility of horizontal acquisition/transmission and exchange between compatible *E. coli* subtypes [42].

This metabolite induces double-stranded DNA breaks in eukaryotic cells, causing cell cycle arrest at the G_2_-M phase and chromosomal aberrations [43].

Colibactin biosynthesis starts from an assembly line machinery located in the *pks* genomic island (54 kb,) which consists of 19 genes comprising non-ribosomal peptide megasynthases (NRPS; *clbH*, *clbJ*, and *clbN*), polyketide megasynthases (PKS; *clbC*, *clbI*, and *clbO*), two hybrid NRPS-PKS (*clbB* and *clbK*), and nine accessory and tailoring enzymes [43]. A recent study has shown that the production of colibactin’s precursor, precolibactin1489, requires every biosynthetic gene in the colibactin gene cluster, and colibactin is formed through the union of two complex biosynthetic intermediates. This coupling generates a nearly symmetrical structure that contains two electrophilic cyclopropane warheads [44]. *Pks*-positive *E. coli* and a synthetic compound carrying a cyclopropane ring system, similar to that detected in precolibactin candidates, were recently shown to alkylate cellular DNA or purified plasmid DNA, respectively, resulting in extensive DNA fragmentation [45,46].

Colibactin-producing bacteria have the capacity to protect themselves from the DNA-damaging action of this metabolite through the expression of ClbS, which deactivates colibactin and confers resistance to its genotoxic effect [47].

The processes of uptake and internalization of colibactin into host cells are still largely unknown, but intoxication requires direct contact between the *pks*-positive bacterium and the target cell [43,48]. Additionally, the ultimate steps of colibactin’s intracellular trafficking and nuclear access have not been yet identified.

#### 2.1.4. DNA Damage Response

Bacterial genotoxins target the nuclei of host cells, where they inflict their DNA damaging activity. Upon intoxication, eukaryotic cells perceive the damage and initiate the classical DNA damage response (DDR), whereby the cell cycle progression is blocked and repair mechanisms are activated. CDT and TT have the capacity to induce double-strand breaks (DSBs) and single-strand breaks (SSBs), while colibactin induces inter-strand cross-links, which can lead to DSBs. Sensing of damage is mediated by three different kinases, depending on the typology of perturbation: the DNA-dependent protein kinase (DNA-PK) and ataxia telangiectasia mutated (ATM), which mainly detect and respond to DSBs, and ATM-and Rad3 related (ATR), which senses SSBs. In the presence of DNA cross-links, both ATM and ATR are activated [49].

ATM and ATR are recruited to the site of the damaged DNA by the Nbs1–Mre11–Rad50 (MNR) complex or the replication protein A (RPA), respectively. Upon autophosphorylation that activates their kinase activities, both kinases initiate checkpoint responses via the CHK2–p53 axis (ATM) and CHK1–CDC25 axis (ATR), resulting in cell cycle arrest and activation of DNA repair processes. Detailed information summarising the DDR response activated by BTGX can be found elsewhere [4].

The repair machinery consists of three main pathways: (i) non-homologous end joining (NHEJ) repair promoted by DNA-PK, which is carried out by ligation of the two broken DNA ends without requiring a repair template; (ii) homologous repair (HR), in which the resynthesis of the damaged region is accomplished using the undamaged sister chromatid as a template [50,51]; and (iii) Fanconi anaemia (FA) pathway to repair DNA inter-strand cross-links. All these systems are activated in response to BTGX intoxication [52,53,54].

### 2.2. Biological Functions of BTGXs

In this section, we describe the effects of genotoxin intoxication with a focus on: (i) the outcome when the DNA damage response fails; and (ii) their novel and mostly unexplored effects as anti-inflammatory mediators.

#### 2.2.1. Unresolved DNA Damage Induced by BTGXs

The cell cycle is a series of events that allows cell division and it consists of four phases, defined as G1, S, G2, and M. The G1 and G2 phases are preparatory stages that precede the S phase (synthesis of DNA) and M phase (mitosis), respectively. At the end of each preparatory phase, there is a checkpoint that ensures that all the requirements are completed in order to progress to the next phase: one at the G1/S phases and another at the G2/M phases. Checkpoints can be activated by different events, with DNA damage being a key inducer of these responses, leading to cell cycle arrest and activation of repair machineries. Repaired cells can subsequently resume cell cycle progression. However, if the DNA damage is beyond repair, cells are either eliminated by apoptosis or enter a permanent dormant state known as senescence, activating a tumorigenesis barrier that prevents the proliferation of cells with the high potential to acquire genomic instability and malignant transformation [9].

We briefly discuss here the main features of apoptosis and senescence induced by BTGX.

##### Apoptosis Induced by Bacterial Genotoxins

The purpose of apoptosis, in this context, is to eliminate defective and damaged cells. Several factors, such as gamma and ultraviolet irradiation, deprivation of growth factors, chemotherapeutic drugs, oncogenes, and viral and bacterial infections, are examples of apoptosis triggering factors [55].

Apoptosis can be initiated by two main mechanisms: the extrinsic pathway that involves death receptor activation (e.g., Fas and TNF receptor 1, TNFR1), leading to activation of caspase 8, and the intrinsic pathway activated by an altered mitochondrial membrane permeability after cellular stress or cellular damage, leading to activation of caspase 9 [56]. Several CDTs (AaCDT, *Helicobacter suis*, HsCDT, and HdCDT) induce apoptosis in key cells of the immune system such as T lymphocytes (both in primary human purified T cells and the established cell lines Jurkat and MOLT-4 [57,58,59,60]) and professional antigen presenting cells, such as macrophages and dendritic cells [61,62,63]. Two major players in the induction of CDT-induced apoptosis are the tumour suppressor protein TP53 (also known as p53) and members of the caspase family [56].

Exposure of lymphocytes to AaCDT induces activation of caspase 9, which is involved in the initiation of apoptosis via perturbation of the mitochondrial function, and possibly caspase 8 via additional activation of death receptors, such as Fas [58,59,60]. AaCDT-induced apoptosis can further depend on the blockage of the PI3K signalling cascade and increased levels of the cell cycle inhibitor p21 [64,65]. Similar patterns of caspase activation have been described for the *Helicobacter hepaticus* HhCDT, which upregulated expression of the pro-apoptotic protein Bax with a concomitant decrease in the anti-apoptotic protein Bcl2. Changes in the expression of these apoptotic key players promote the release of mitochondrial cytochrome C and consequently activate caspases 9, 7, and 3 in epithelial cells, the first line of innate defence against pathogens [66].

Interestingly, AaCDT-mediated cell death in the MOLT-4 cell line can be induced by two different pathways: the conventional caspase-dependent apoptosis in the early phase and a caspase-independent apoptosis-like pathway at later stages. Both require the mitochondrial membrane disruption pathway [60], highlighting the complexity of the intoxication-associated regulation of intracellular responses. Whether these described mechanisms are maintained during in vivo scenarios of infection is poorly understood and will require future studies.

##### BTGXs-Induced Senescence

Upon entry into senescence, cells exhibit characteristic features, such as the formation of promyelocytic leukaemia (PML) nuclear bodies, altered metabolism, and secretion of pro-inflammatory mediators (senescence-associated secretory phenotype, SASP) that remodel the surrounding microenvironment. Even though senescence prevents damaged cells from proliferating, representing a tumorigenesis barrier, long-term senescence is related to chronic inflammation and cancer [9].

Senescence can be exploited by infection agents, as recently reviewed by Humphreys et al. [67]. Induction of senescence, which was transmissible to bystander cells, and secretion of a pro-inflammatory SASP were observed in cells exposed to HdCDT or TT [68,69]. The TT-induced senescence enhanced intracellular *Salmonella* infection in macrophages and fibroblast-like cells [69], highlighting how bacteria may induce and exploit DNA damage-associated senescence to enhance their invasive capacity.

Induction of BTGX-associated senescence was also proven upon in vitro infection of a broad panel of non-transformed and transformed fibroblasts and epithelial cell lines with *pks+ E. coli* [70]. The *pks*-induced senescent cells presented a SASP, characterised by secretion of pro-inflammatory molecules (IL-6, IL-8, MCP1, MMP3), and the senescent features were transmitted to bystander cells [70]. As proof of the SASP’s tumour-promoting activity, colibactin and HhCDT-induced SASP was also demonstrated to be a key factor in promoting tumour growth in xenograft mouse models [71,72,73].

#### 2.2.2. Acquisition of Genomic Instability

Genomic instability refers to increased frequencies of genetic alterations and base pair mutations in host cells, and is one of the enabling characteristics of cancer [74].

Induction of genomic instability by long-term exposure to sublethal doses of BTGXs has been extensively studied and demonstrated in experimental models in vitro, highlighting the relevance of the exposure dose and time on the definition of the intoxication outcome.

In vitro exposure to sublethal doses of HdCDT and HhCDT supported malignant cell transformation by increasing the mutation frequency, accumulating chromosomal aberrations and promoting anchorage-independent growth [75]. Furthermore, prolonged infection of Chinese Hamster Ovary (CHO) cells with low doses of *pks*+ *E. coli* revealed a higher percentage of phosphorylated histone H2AX (γH2AX) foci in cell nuclei, as an indicator of DNA damage [76]. Interestingly, unrepaired DSBs resulted in chromatin bridges during the anaphase, lagging chromosomes, and multipolar mitosis, as well as aneuploidy and tetraploidy [76]. Recent studies on colon organoids have shown that short-term infection with *pks*+ *E. coli* promoted the generation of organoids that, due to endogenous Wnt production, which is normally required for cell determination and proliferation [77], grew independently of Wnt supplementation in the culture medium [78], thus resembling aberrant Wnt signalling, which is observed in the majority of colorectal carcinoma (CRC) patients [79,80]. Interestingly, the Wnt-independent organoids displayed a higher mutation load and more chromosomal aberrations [78]. This study highlighted the transforming capacities of *pks*+ *E. coli* during early malignant transformation, emphasising the molecular mechanisms that link colibactin-producing bacteria and DNA damage, and supporting their potential carcinogenic properties.

The contribution of genotoxin-producing bacteria on carcinogenesis has been described only in inflammatory or genetic cancer-related in vivo models. Infection of colitis-susceptible *Il10*-deficient (*Il10*^-/-^) mice with CDT-deficient *H. hepaticus* or *H. cinaedi* resulted in less severe typhlocolitis (inflammation of the caecum and adjacent colon) compared with infection with the CDT wild-type isogenic strains [81]. In addition, colonization with the CDT-proficient *H. hepaticus* or *H. cinaedi* caused intestinal dysplasia and intramucosal carcinoma in the *IL10^-/-^* and *Rag2^-/-^* (T and B cell deficient) models [82,83] or liver dysplasia in Swiss Webster mice [84].

Several studies in cancer-prone models have been also performed to assess the carcinogenic potential of *pks*+ *E. coli*. Administration of the pro-carcinogenic agent azoxymethane (AOM, which mimics sporadic CRC) in germ-free *Il10*^-/-^ mice showed an enhanced development of adenocarcinomas without affecting inflammation upon monocolonisation with the commensal *pks*+ *E. coli* strain NC101 [85]. The pro-inflammatory-prone intestinal environment of *Il10*^-/-^ mice further favoured the expansion and maintenance of *Enterobacteriaceae* compared with healthy wild-type mice [86].

Interestingly, in this context, *E. coli*, *Bacteroides fragilis, Fusobacterium nucleatum*, *Parvimonas micra*, and *Peptostreptococcus stomatis* are enriched in biofilms identified in patients with familial adenomatous polyposis (FAP), a hereditary condition caused by germline mutations of the adenomatous polyposis coli (APC) tumour suppressor gene, frequently mutated in CRC [87,88,89].

Synergy in colon carcinogenesis between the *pks*+ *E. coli* and *B. fragilis* has been demonstrated in two models of sporadic CRC (by administration of AOM) and hereditary CRC (the Apc Min^Δ716/+^ mouse model), further corroborating the relevant role of colibactin in colon tumorigenesis [89]. The higher abundance of colibactin-producing bacteria in patient samples from inflammatory bowel disease (IBD) and the most aggressive forms of CRC clinically confirmed the relevance of the in vitro and in vivo findings [85,90].

*Campylobacter* species have also been found to be enriched in CRC samples [91,92], and studies on germ-free mice haplo-insufficient for the APC gene (Apc^Min/+^) subjected to dextran sodium sulphate (DSS) reported the development of large tumours in the distal colon of mice infected with a *C. jejuni* strain carrying a functional CdtB subunit [93].

#### 2.2.3. Immunomodulatory Properties of BTGX

Considering that DNA damage is regarded as a danger for the cell homeostasis, it is expected that infection with genotoxin-producing bacteria will promote a pro-inflammatory response. Indeed, several studies have demonstrated the pro-inflammatory effects of BTGX, such as Hh-CDT, Hp-CDT, Aa-CDT, CjCDT, and colibactin-producing *E. coli* in vitro [32,94,95,96,97,98,99,100] and in vivo [82,83,84,101,102,103,104,105,106].

The DNA-damage induced pro-inflammatory response can be activated by four pathways, which are not mutually exclusive: (i) NFκB activation [104], possibly via the ATM kinase pathway [7]; (ii) mitogen-activated protein kinase (MAPK), p38, and signal transducer and activator of transcription (STAT3) phosphorylation [83,107], which lead to the production of pro-inflammatory cytokines, IL-1β, IL-6, IL-8, and TNF-α [108,109,110]; (iii) formation of by-products of mitotic progression (micronuclei), which activate cGAS-STING signalling to further secrete Type I interferons [111,112,113,114]; and (iv) secretion of SASP, characterised by IL-1α, IL-6, IL-8, MMP-1, and MMP-3 [9,68,69,70]. Together, these secreted pro-inflammatory cytokines further activate pro-inflammatory and efficient anti-bacterial T helper (Th)1 and Th17 immune responses [94,115,116,117]. The underlying activation mechanisms of these pathways have been recently reviewed by Martin and Frisan [4], and will not be described here in detail.

Here, we will focus on describing the anti-inflammatory and immunosuppressive roles of BTGXs, which have not been as widely reviewed.

It has been shown that both AaCDT and HdCDT block the proliferation of T lymphocytes by inducing G2/M cell cycle arrest and apoptosis, suggesting an immunosuppressive role of these toxins [57,118]. Furthermore, *pks+* extraintestinal *E. coli* (ExPEC) induced DNA damage, cell cycle arrest in the G2/M phase, and cell death in T lymphocytes in vitro [119]. Induction of cell death and cell cycle arrest in lymphocytes may significantly affect the course of infection, favouring the bacterial dissemination, multiplication, and persistence of BTGX-carrying pathogens [120]. Thus, the immunosuppressive effect of colibactin may explain the increased mortality induced by infection with a *pks*+ *E. coli* compared with an isogenic colibactin-deficient strain (Δ*clbA*) strain in a mouse model of sepsis [119].

The anti-inflammatory effect of BTGXs has been also shown for the *pks*+ probiotic *E. coli* strain Nissle 1917, where deletion of the *cblA* gene abolished the genotoxin effect in vitro, as well as the anti-inflammatory properties of this strain in two independent models of colitis [121]. However, considering the complexity of the *pks* island, to date, it has not been possible to define unequivocally whether the immunosuppressive effect of the Nissle strain is directly mediated by the DNA-damaging activity of colibactin. Therefore, additional experiments including mouse models deficient in a functional DDR, such as ATM-deficient mice, could help to address this important and interesting question, thereby shedding light on the underlying mechanisms involved.

Infection studies performed with a TT-expressing *S. enterica* have shown the suppression of gastroenteritis characterised by a decrease in the recruitment of CD45^+^ (leukocytes), CD3^+^ (T lymphocytes), and F4/80^+^ (macrophages) cells [122,123,124]. The presence of a functional TT promoted the induction of a Th2 tissue-protective immune response characterised by the presence of anti-inflammatory CD206^+^ macrophages and T regulatory lymphocytes, and enhanced expression of mRNA for Th2 cytokines (*Il10*, *Il4*, *Il13*, *Il5*) [124]. These anti-inflammatory effects were partially ATM-dependent and occurred in spite of the significant induction of DNA fragmentation in the colon mucosa and the substantial presence of senescent cells, supporting the idea that, under these conditions, DNA damage and senescence are uncoupled from inflammation.

Interestingly, a functional TT did not prevent inflammation in the liver and spleen [122], and the anti-inflammatory effects were lost when infection occurred in mice suffering from acute DSS-induced colitis [124]. Therefore, the described effects of a functional TT were context- (healthy vs. colitis) and tissue- (intestine vs. liver and spleen) dependent.

## 3. Pore-Forming Toxins

### 3.1. General Overview and Mode of Action

There are different types of PFTs secreted by many Gram-positive and Gram-negative bacteria, which strengthens the relevance of these effectors in the interaction of bacteria with the surrounding microenvironment by targeting an essential structure of prokaryotic and eukaryotic cells: the membrane. Many PFTs are secreted as monomers by the producing bacterium and oligomerise within the plasma membrane of target cells, evoking the formation of pores with different sizes: large pores in the range of 20–200 nm and small pores in the range of 0.5–5 nm. PFTs are further classified based on the secondary structure of the region that is inserted into the host cell membrane, forming either α-helices (α-PFTs) or β-barrels (β-PFTs). PFTs bearing the latter include the large-pore-forming cholesterol-dependent cytolysins (CDCs), produced by Gram-positive and some Gram-negative bacteria [2]. The small-pore-forming repeat in toxin (RTX) toxins, produced by Gram-negative bacteria, form an additional large group of PFTs [2,5,125].

Beside these PFT families, the binding (B) component of some multi-subunit AB toxins can form pores in the plasma or endosomal membrane to allow the internalisation of the active (A) subunit, and the tip of Type III and IV secretion systems penetrates bacterial or cellular membranes as a pore-forming-like structure to allow translocation of effector proteins [5].

In this review, we will focus on α-PFTs, β-PFTs and RTX. Considering the great variability of PFTs and the complexity of their interaction with the plasma membrane, we mainly discuss the PFT-induced modulation of key signalling pathways that are associated with the host immune response, with a specific focus on the immunosuppressive effects, which are independent of the killing of immune cells. For a more detailed overview of PFTs, their structures, types of pore, modes of action, and size-dependent repair mechanisms, we refer the reader to excellent reviews [2,5,126].

### 3.2. PFT-Mediated Modulation of Intra-Signalling Pathways

The cellular responses to PFTs are highly variable, ranging from cell death due to necrosis, pyroptosis, or apoptosis to modulation of diverse intracellular signalling pathways [2,5]. The final outcome depends on a combination of multiple factors such as pore size, the amount of toxin produced, the site of production, the microenvironment gradient, membrane stiffness, and cell type.

Necrosis and pyroptosis are the results of loss of membrane integrity and are induced either by the direct effect of the PFT or via activation of the inflammasome-dependent cellular pore-forming protein Gasdermin D [127]. Paradoxically, this can promote two antagonistic effects: (i) it favours bacterial invasion, via the destruction of barrier integrity and immune evasion, due to specific killing of leukocytes, the first line of innate immune defence; (ii) it activates inflammation via release of DAMPs and inflammasome activation, alerting and promoting the host immune response [2,128].

Beside cell death, PFTs induce other types of cellular responses, which range from pore-size-dependent membrane repair [5,129], regulation of transcription factors, and, consequently, a variety of intracellular signalling pathways, to post-translational modifications (e.g., sumoylation) that have broad effects in an autocrine and paracrine manner. In this section, we mainly highlight the key events that have a direct impact on the immunomodulatory activity, namely alteration of ion concentrations and modulation of the activity of key protein kinases.

#### 3.2.1. Alteration of Ion Concentrations

Many of these effects are associated with the alteration of ion concentrations within the cell, such as calcium (Ca^2+^) influx from the extracellular environment and potassium (K^+^) efflux from the cytosol. The extent of the ion imbalance depends on the pore size, as well as the number of pores per cell, enabling either rapid and sustained Ca^2+^ influx and K^+^ efflux [5] or a more sophisticated alteration of intracellular ion homeostasis. Indeed, sublethal amounts of small pore-forming toxins such as the *E. coli* alpha haemolysin (HlyA) and cytolysin (ClyA), the latter delivered via OMVs [130], induce an oscillatory pattern in the intracellular Ca^2+^ concentration, albeit different in the oscillation periodicity [131,132]. The HlyA-induced oscillations are dependent on Ca^2+^ influx mediated by voltage-operated L-type voltage Ca^2+^ channels and released from endoplasmic reticulum stores by inositol triphosphate. This triggers activation of the pleiotropic transcription factor NFκB, resulting in secretion of the pro-inflammatory cytokines IL-6 and IL-8 [131]. Conversely, ClyA-dependent oscillations are independent of extracellular Ca^2+^ and rely on Ca^2+^ release from intracellular stores, which does not induce the secretion of IL-8 [132].

But why do PFTs induce different types of Ca^2+^ mobilisation, leading to distinctive cellular responses? A possible explanation might be the different interactions of the toxins with the host cells. HlyA is released as a soluble toxin and interacts with a membrane receptor, as demonstrated by the use of the receptor agonist 6-cyano-7-nitroquinoxaline-2,3-dione (CNQX) [131]. Instead, ClyA is delivered via OMVs [130], and it is assumed that this delivery mode induces the internalisation of the toxin-loaded vesicle. Thereby, the toxin bypasses the interaction with the plasma membrane, allowing direct access to components of the intracellular compartment. In addition, HlyA is encoded by pathogenic *E. coli* (UPEC) strains [133], while ClyA can also be expressed by laboratory non-pathogenic strains, such as K12 [134]. Therefore, the different profile of pro-inflammatory mediators induced by the two PFTs may depend on the specific niche of bacterial colonisation and the microenvironment. Moreover, the route of delivery, as well as the level of toxin produced or delivered into host cells, might strongly influence the ion homeostasis of the host cell, resulting in different outcomes of infection.

Induction of Ca^2+^ oscillation can also be induced by sublethal concentrations of the large pore-forming CDCs such as listeriolysin O (LLO), a key virulence factor in *Listeria monocytogenes* [135], which is dependent on Ca^2+^influx from the extracellular environment, as well as release from intracellular stores [136,137]. The latter involves channel-independent mechanisms and phospholipase C (PLC) inositol 1,4,5-trisphosphate receptor (IP_3_R)-operated Ca^2+^ channels activated via G-proteins and protein tyrosine kinases [137]. The impact of LLO on the intracellular Ca^2+^ concentration resembles that modulated by sublethal doses of aerolysin, produced by the pathogenic bacteria *Aeromonas hydrophila* [138].

As discussed in the next section, the alteration of the Ca^2+^ homeostasis induced by LLO and aerolysin has a great impact on the induction of the inflammatory or tolerogenic states in different cells of innate and adaptive immunity. Thus, PFT-induced intracellular signalling modulates not only the initial inflammatory response but also influences the adaptive response (tolerogenic vs. non-tolerogenic), which has a relevant impact on the establishment of chronic infections [3].

#### 3.2.2. Alteration of Protein Kinase Activity

Other early events associated with PFT–host cell interactions are the activation or inhibition of kinases such as MAPKs (p38 MAPK, JNK, and ERK) and the protein kinase AKT, respectively [139]. These kinases have pleiotropic effects on cell signalling, ranging from activation of survival signals to regulation of inflammatory responses. With *Caenorhabditis elegans* as a model organism, both p38 MAPK and JNK have been shown to be central nodes in the activation of defence responses to PFT exposure. The main scope of these signalling pathways is to control: (i) processes that promote the recovery of homeostatic perturbations induced by PFTs (e.g., restoration of the ion balance and the plasma membrane integrity), and (ii) activation of innate immune responses by orchestrating the release of the pro-inflammatory cytokines [140,141,142,143,144,145]. In light of the diversity of pore sizes and the complex interactions with the host membranes, it is worth highlighting that MAPK activation does not always promote beneficial effects for the target cells. Thus, pharmacological inhibition of p38 MAPK abolishes the recovery from intoxication with the *Staphylococcus aureus* α-toxin in HaCaT cells, while the same treatment does not affect cells exposed to streptolysin O (SLO) [146].

Bacteria expressing HlyA, aerolysin, or α-toxin at sublytic toxin doses strongly inhibited the activation of the AKT kinase in human bladder epithelial cells. This effect could not be overcome by treatment with powerful activators of AKT, such as epithelial growth factor (EGF) or TNFα [147]. AKT activation promotes the signalling pathways regulating cell growth, survival, and—importantly within the context of this review—inflammation via inducing the gene expression of pro- and anti-inflammatory cytokines, regulating macrophage polarisation and enhancing neutrophil degranulation and phagocytosis [148,149].

### 3.3. Immunosuppressive and Tolerogenic Effect of PFTs

Considering that the immune system has evolved to perceive and eliminate threats that might be harmful to the organism, it is not surprising that the presence of a toxin which damages cellular membranes evokes an immune response and cellular repair mechanisms [5,11]. A less explored facet of PFTs is their immunosuppressive and tolerogenic effects, which can be viewed from the perspective of a subtle remodelling of the host’s response to avoid complete bacterial clearance by the immune response and concomitantly limiting the tissue damage to the host. This delicate balance may allow the microbe to establish a suitable niche for replication and spreading, possibly leading to a persistent asymptomatic infection: the ideal condition for a pathogen (Figure 1).

In this section, we highlight the key mechanisms by which PFTs have been shown to modulate the immune response of the host towards a tolerogenic microenvironment (Figure 3).

#### 3.3.1. Inhibition of Pro-Inflammatory Cytokine Release

Sensing a threat via DAMPs or PAMPs results in the production of pro-inflammatory cytokines. These cytokines alert and activate cells of the innate immunity that represent the first line of defence and orchestrate the activation of the most suitable adaptive immunity to eliminate the threat [117,150]. Therefore, it is not surprising that several PFTs have been shown to interfere with this initial key step. In 1993, it was demonstrated that the presence of HlyA strongly reduces the induction of the proinflammatory cytokines IL-1β, IL-6 and TNFα at the mRNA and protein levels in a mixture of human lymphocytes, monocytes, and basophils (LMB), counterbalancing the strong pro-inflammatory effect evoked by the bacteria fimbriae. This effect did not correlate with HlyA-induced cell death, since viability was not impaired [151].

Interestingly, Bhushan and colleagues showed that exposure to purified HlyA or *E. coli* expressing a functional *hlyA* gene induced a differential response in rat peritoneal (PM) and testicular macrophages (TM) upon stimulation with LPS, with downregulation of *Il1α*, *Il1β,* and *Il6* in PM, and upregulation of *Il1β* and *Il6* in TM [152]. Both cell types exhibited increased levels of *Il4* and *Il13* mRNA, two Th2 anti-inflammatory cytokines [153]. The distinct cytokine profiles were dependent on the activation of different transcription factors in PMs and TMs: the Ca^2+^-dependent nuclear factor of activated T cells (NFAT) only, or NFAT and the MAPK-regulated activator protein 1 (AP1), respectively [152]. Interestingly, MAPK activation, inhibition of IL-12 and nitric oxide synthase 2 (NOS2) (a pro-inflammatory and anti-bactericidal molecule, respectively), and induction of IL-10, an anti-inflammatory Th2 cytokine, was also observed in response to exposure of murine bone-derived macrophages (BMDMs) to the Group B *Streptococcus* (GBS) beta hemolysin/cytolysin (h/c), the Group A *Streptococcus* (GAS) streptolysin (SLO), and LLO [154]. These data highlight that MAPKs and NFAT are key nodes in the PFT-mediated immunoregulation of macrophages.

Another mechanism by which PFT can suppress pro-inflammatory cytokines is the promotion of proteolytic degradation by hijacking the ubiquitin proteasome system. This has been demonstrated in murine BMDMs exposed to SLO. The GAS strain 854 is able to induce ubiquitination and reduction of pro-IL-1β levels, which is normally processed into its biologically active form by activated inflammasomes [155]. The ubiquitination of pro-IL-1β was observed only when cells were co-cultured with the live bacterium, producing a SLO form that can insert into the host membrane and form pores, but was completely lost when an active recombinant toxin was used. Thus, a pore-forming SLO was necessary but not sufficient to induce this suppressive effect, indicating that additional bacterial product(s) were required to promote pro-IL-1β ubiquitination [155].

Suppression of pro-inflammatory cytokines can also be a direct consequence of the membrane repair pathways through membrane vesicle shedding, leading to reduced levels of membrane proteins, including key pathogen recognition receptors (PRRs), such as Toll-like receptor (TLR) 4, the downstream adaptor MyD88, and the interferon Type II receptor (IFNγR1). Thereupon, BMDMs exposed to functional SLO and PFO show lower levels of TNFα induction upon challenge with the TLR4 agonist, lipopolysaccharide (LPS), which is present on the outer membrane of Gram-negative bacteria [156]. The secretion of IFNγR1 via the vesicles may also account for the significant reduction of the co-stimulatory molecule CD80, which is a key molecule involved in the activation of naïve T cells [157].

The multifunctional-autoprocessing repeats-in-toxin (MARTX) toxins produced by several *Vibrio* species represent an ingenious evolutionary solution because these pore-forming toxins translocate several domains that alter the host’s barrier integrity, allowing bacterial invasion, and simultaneously silence the inflammatory response of epithelial cells [158]. For further information on these interesting multidomain toxins, which are not exclusively pore-forming toxins, we refer the reader to Kim et al. [159].

As a conclusion of this section, there are several reflections that need to be highlighted: (i) several bacterial factors can contribute to the effect of PFTs on the regulation of the host’s immune response, indicating the importance of studying the effects of the whole bacterium vs. the purified toxin; (ii) PFT may have differential effects on different cell subpopulations even within the same cell lineage (e.g., macrophages).

#### 3.3.2. Polarization of the Host Immune Response

The induction of a tolerogenic immune response has been demonstrated for several PFTs, and ranges from modulating macrophages’ polarisation to induction of anergy in T lymphocytes, as outlined below.

*S. pneumoniae* is a colonizer of the upper respiratory tract of healthy individuals but is also the cause of severe diseases ranging from pneumonia to meningitis. PLY is a key virulence factor [160], and it has been recently shown that infection of primary human monocyte-derived dendritic cells (DCs) with the pneumococcal strain T4R significantly reduced the secretion of pro-inflammatory cytokines (TNFα, IL-1β, and IL-12) compared with an isogenic strain carrying a deletion of the *ply* gene. This effect was associated with an upregulation of the suppressor of cytokine signalling (SOCS) 1 at both the mRNA and protein levels, downregulation of the NFκB subunit p65, and reduced phosphorylation of the transcription factor STAT1. This response was mediated by the interaction of PLY with the mannose receptor C type 1 (MRC-1), an interaction that further promoted bacterial internalisation into non-lysosomal compartments [161]. Interestingly, DCs exposed to the PLY-producing T4 strain polarised naïve T cells toward an IFNγ-low, IL-4-high and Forkhead box p3 (FOXP3)-positive phenotype, features of regulatory T lymphocytes (Treg) [161], which have immunosuppressive activity [162].

Polarisation of immune cells can also be mediated through membrane microvesicles secreted by cells exposed to PLY-producing *S. pneumoniae* as consequence of the intrinsic cell repair mechanism [5]. Microvesicles purified from epithelial HEK293 cells exposed to PLY can induce polarisation of human macrophages into a specific subpopulation, which retains the ability to secrete IL-6 but expresses lower levels of the major histocompatibility complex (MHC) Class II and the co-stimulatory molecule CD86 [163], two key factors essential for T lymphocyte activation [157,164]. The MHCII^low^CD86^low^ phenotype is reminiscent of the anti-inflammatory M2-like macrophages, although no specific M2 markers (CD206, CD163) were upregulated in this set of experiments. [163]. Liposomes loaded with EGPF-tagged PLY were internalised by purified macrophages and induced a similar phenotype. These macrophages showed significantly impaired TNFα release in response to LPS, while secretion of IL-6 was not affected [163].

Re-polarisation of dendritic cells is a strategy used by *Helicobacter pylori* to remodel the host’s response and establish a persistent infection in the gastric mucosa that is associated with gastritis and a high risk of developing cancer. Interestingly, the presence of this bacterium also suppresses allergen-induced asthma in experimental mouse models [165]. Persistent infection with this bacterium is dependent on the expression of the pore-forming toxin VacA, as demonstrated by use of an in vivo colonization model using an *H. pylori* PMSSS1 Δ*vacA* strain and its isogenic wild-type control. This effect was directly dependent on VacA’s ability to direct the polarisation of DCs, key antigen presenting cells (APC) for T cell activation, towards a tolerogenic phenotype that stimulates FOXP3 and CD45-positive Treg lymphocytes, and prevents the development of the strong adaptive inflammatory response mediated by Th1 and Th17 [117]. VacA has been shown to interfere with cytokine secretion by DCs, thereby preventing the upregulation of the co-stimulatory molecules CD80 and CD86, and secretion of the Th1-polarizing cytokines IL-12 and IFNγ upon LPS stimulation or exposure to the Gram-positive bacterium *Lactobacillus acidophilus* [166,167]. The direct demonstration that this PFT is essential for maintaining a delicate balance between reducing bacterial clearance and limiting tissue immunopathology is demonstrated by the high degree of gastric pathology (including gastric atrophy, hyperplasia, and metaplasia) in mice infected with a Δ*vacA* strain [166]. It would be very interesting to unravel the underlying molecular mechanisms that coordinate DC polarisation and tightly control the delicate switch towards the tolerogenic state.

The immunomodulatory effects of VacA are multifaceted and can affect other aspects related to activation and regulation of the host’s adaptive immunity. These include the inhibition of antigen presentation from newly-synthesised MHC Class II molecules [168], as well as direct T cell activation [169]. The former may be dependent on the VacA-induced membrane damage of intracellular vesicular compartments, leading to alteration of the ion concentrations and insufficient acidification of the MHC II compartment, where antigen degradation and peptide loading on the MHC II molecules occur [168]. The latter is due to VacA-mediated interference in the signalling pathway induced by IL-2 (the key T lymphocyte growth factor [169]) by abrogating Ca^2+^/calcineurin-dependent activation of the transcription factor NFAT in the T cell line Jurkat [170].

Similar effects on the proliferation of CD4^+^ T lymphocytes were observed in response to LLO-producing *L. monocytogenes* or purified toxin. However, contrary to the previous data, the T cell unresponsiveness was associated with the activation of NFAT and possibly NFAT-regulated genes that impaired signalling from the T cell receptor (TCR) [171], inhibiting the first step of T cell activation [172].

An interesting aspect is that VacA or LLO activated or inactivated NFAT, respectively, though leading to the same outcome: inhibition of T cell proliferation. These results may depend on the different cells used, such as the leukemic T cell line Jurkat [170] vs. normal murine T lymphocytes [172], as well as on the different types of stimulus used for T cell activation: phytohemagglutinin (PHA) and phorbol myristate acetate (PMA) for Jurkat [170], or immunisation with ovalbumin in vivo or anti-CD3 antibody plus PMA and ionomycin in vitro for the mouse T lymphocytes [172]. It may also reflect the activation of different pathways by the two toxins, possibly due to interactions with membrane-bound host protein(s).

Induction of cell anergy of pro-inflammatory immune cells such as mast cells can also be modulated by PFT. Specifically, prolonged exposure to LLO has been shown to prevent mast cell activation by IgE cross-linking due to depletion of intracellular Ca^2+^ stores [173].

Overall, these data indicate that the immunomodulatory effects of PFT may be exerted at very early stages of the microbe–host interaction by targeting pleiotropic effectors such as NFAT, MAPKs, and NFκB, while longer-term exposure results in the exhaustion of cellular responses.

Infections occur in the context of complex niches where the pathogens interact with the host microenvironment and the local microbiota. Therefore, it is not surprising that PFTs can influence the outcome of other types of infections by acting as immunomodulators. This was shown in a mouse model of infection with influenza A virus (A/PR/8/34), in which mice were pre-colonised for 10 days with the PLY-producing *S. pneumoniae* strain P1121 (serotype 23F) prior to infection with influenza A virus. The authors showed that pre-colonisation with the PLY-proficient bacterium prevented a strong tissue-destructive inflammatory response in the lungs, and weight loss. This effect was dependent on the PLY-mediated induction of a subset of anti-inflammatory alveolar macrophages with a M2-like phenotype (CD206 and arginase positive) and was lost when *S. pneumoniae* was administered after the viral infection, which, conversely, resulted an increase in the severity of the disease [174].

#### 3.3.3. Enhanced Colonisation

The relevance of the microenvironment in the regulation of the host’s immune responses to determine the delicate balance between bacterial asymptomatic colonisation and pathogenesis is supported by the role of *S. aureus* toxins, many of them acting as PFT (α-toxin, β-toxin, LuvAB, LukED, and LukSF). A study comparing *S. aureus* isolates belonging to the ST15 lineage from a human asymptomatic nasal carrier over a 12 month-period and bloodstream isolates collected after an episode of bacteraemia at month 15 showed that toxin expression (leading to high toxicity isolates in vitro) was a common trait in the nasal carriage stage, while bacteraemia and increased fitness to the hostile environment, represented by the human serum, favoured the presence of isolates with reduced lytic activity (in vitro) [175]. This is in line with a demonstration that mutations within the gene encoding the transcription factor repressor of surface proteins (*rsp*) promoted the appearance of a low cytotoxicity non-haemolytic *S. aureus* capable of bloodstream infections [176]. Thus, the cost of toxin production may strongly impact the fitness of *S. aureus* to survive in hostile environment but may favour colonisation and transmission. It is also possible that the expression of *S. aureus* α-toxin enhances nasal colonisation via biofilm formation, since it has been shown in vitro that expression of this toxin contributes to biofilm formation in static conditions and well-structured adhesive microcolonies under flow conditions [177].

What would be very interesting, although technically difficult, is to assess how much leukotoxins are produced in the nasal environment of asymptomatic carriers, whether their expression is regulated by the local microbiota, whether toxin expression promotes the acquisition of a tolerogenic immune response to favour the status of an asymptomatic carrier (see previous section), and whether PFT-induced biofilm formation also occurs in the context of the complex mucosa environment of the nasopharynx.

### 3.4. BTGX and PFT-Like Protein in the Animal Kingdom

At the end of this journey, we found it quite important to devote few sentences to the remarkable findings showing that a homologue of the *cdtB* gene subunit of CDTs is also found in insects, while aerolysin-like proteins (ALPs) are found in vertebrates [178], highlighting the biological relevance of these effectors outside the Monera kingdom.

A recent study has identified the presence of the *cdtB* gene in several species of vinegar flies and aphids [179]. The gene found in these insects is closely related to an orthologue found in the bacterial symbiont of aphids (*Candidatus* Hamiltonella defensa) [180] and highlights the horizontal gene transfer of this CDT component. Based on phylogenetic analysis, this gene has been maintained in insects for millions of years, suggesting an evolutionary advantage for the eukaryotic animal. The *cdtB* gene, cloned from *Drosophila ananassae,* was expressed as HIS-tagged protein in *E. coli* Rosetta 2(DE3)pLysS. The purified protein was able to induce relaxation and linearization of a supercoiled plasmid in an in vitro assay, similar to the CdtB subunit of *E. coli*, indicating that it retained its genotoxin activity [179]. The interesting and puzzling question that still needs to be addressed is what the function of this gene in metazoans is, and how is it delivered into target cells in the absence of the two binding subunits CdtA and CdtC.

On its skin, the frog *Bombina maxima* secretes the protein βγ-CAT, which is a complex of an aerolysin domain and a trefoil factor. Interestingly, it has been shown in several mouse models of skin injury that βγ-CAT accelerated wound healing by increasing the rate of epithelialisation. This was accompanied by an increase of macrophages with a M2 phenotype (CD206-positive) at the wound site and a rapid release of IL-1β and TGFα [181]. Thus, this PFT-like toxin in frogs exerts two beneficial roles: (i) it promotes a Th2 immune response that favours wound healing [182] and (ii) it activates a rapid inflammatory response that may eliminate potential threats, such as invading microbes [117,150].

## 4. Concluding Remarks

In this review, BTGXs and PFTs have been used as models to highlight that there is a spectrum of outcomes during infections in the host, ranging from killing to a refined hijacking of the tolerance mechanism [16], where the same molecule can act as a natural born killer or a negotiator. This paradox can be reconciled by thinking in terms of the regulation of the toxin expression and the amounts secreted into the microenvironment, the duration of exposure, the complexity of the niche, the interplay with the host and the microbiota, and the status of the host cell(s).

The exponential technological development of new tools to perform in situ multiplex analyses of tissue samples, such as bacterial transcriptomic analysis from in vivo infections, and the use of complex organoid culture systems will allow the scientific community to keep investigating the gold mine of the bacterial toxin realm and learn more about bacterial and host physiology and pathophysiology.

Thus, to honour the 700-year death anniversary of Dante Alighieri (1321), we can say that the immunomodulatory effects of the bacterial toxins discussed in this review resemble the Purgatory cantica: the intermediate step during Dante’s journey from Hell to Heaven described in the *Divina Commedia*: a perfect analogy for biology, where nothing is only black or white but has a beautiful gradation of colours.

## Figures and Tables

**Table 1:** Bacterial genotoxins.

Bacterium	Toxin
*Aggregatibacter actinomycetemcomitans*	AaCDT
*Campylobacter jejuni*	CjCDT
*Escherichia coli*	EcCDT
*Escherichia coli*	Colibactin (*pks*)
*Haemophilus ducreyi*	HdCDT
*Helicobacter cinaedi*	HcCDT
*Helicobacter hepaticus*	HhCDT
*Helicobacter pullorum*	HpCDT
*Helicobacter suis*	HsCDT
*Salmonella enterica*	Typhoid toxin (TT)

**Table 2:** Pore-forming toxins.

Bacterium	Toxin	Acronym
*Aeromonas hydrophila*	Aerolysin	
*Clostridium perfringens*	Perfringolysin	PFO
*Escherichia coli*	Hemolysin	HlyA
*Escherichia coli*	Cytolysin	ClyA
Group A *Streptococcus* (GAS)	Streptolysin	SLO
Group B *Streptococcus* (GBS)	Hemolysin/cytolysin	bh/c
*Helicobacter pylori*	Vacuolating toxin A	VacA
*Listeria monocytogenes*	Lysteriolysin O	LLO
*Staphylococcus aureus*	α-Toxin	
*Streptococcus pneumoniae*	Pneumolysin	PLY
*Streptococcus suis*	Suilysin	SLY

## References

[1] Frisan T., Guidi R., Guerra L., Locht C., Simonet S. (2012). Toxins acting on intracellular targets: Only foes or also friends?. Bacterial Pathogenesis: Molecular and Cellular Mechanisms.

[2] Los F.C.O., Randis T.M., Aroian R.V., Ratner A.J. (2013). Role of Pore-Forming Toxins in Bacterial Infectious Diseases. Microbiol. Mol. Biol. Rev..

[3] Monack D.M., Mueller A., Falkow S. (2004). Persistent bacterial infections: The interface of the pathogen and the host immune system. Nat. Rev. Microbiol..

[4] Martin O.C.B., Frisan T. (2020). Bacterial Genotoxin-Induced DNA Damage and Modulation of the Host Immune Microenvironment. Toxins.

[5] Bischofberger M., Iacovache I., van der Goot F.G. (2012). Pathogenic Pore-Forming Proteins: Function and Host Response. Cell Host Microbe.

[6] Grasso F., Frisan T. (2015). Bacterial Genotoxins: Merging the DNA Damage Response into Infection Biology. Biomolecules.

[7] McCool K.W., Miyamoto S. (2012). DNA damage-dependent NF-kappaB activation: NEMO turns nuclear signaling inside out. Immunol. Rev..

[8] He S.H., Sharpless N.E. (2017). Senescence in Health and Disease. Cell.

[9] Gorgoulis V., Adams P.D., Alimonti A., Bennett D.C., Bischof O., Bishop C., Campisi J., Collado M., Evangelou K., Ferbeyre G. (2019). Cellular Senescence: Defining a Path Forward. Cell.

[10] Murao A., Aziz M., Wang H., Brenner M., Wang P. (2021). Release mechanisms of major DAMPs. Apoptosis.

[11] Jing W.D., Lo Pilato J., Kay C., Man S.M. (2021). Activation mechanisms of inflammasomes by bacterial toxins. Cell. Microbiol..

[12] Soderblom T., Laestadius A., Oxhamre C., Aperia A., Richter-Dahlfors A. (2002). Toxin-Induced calcium oscillations: A novel strategy to affect gene regulation in target cells. Int. J. Med. Microbiol..

[13] Nhieu G.T., Clair C., Grompone G., Sansonetti P. (2004). Calcium signalling during cell interactions with bacterial pathogens. Biol. Cell.

[14] Spaan A.N., van Strijp J.A.G., Torres V.J. (2017). Leukocidins: Staphylococcal bi-component pore-forming toxins find their receptors. Nat. Rev. Microbiol..

[15] Tromp A.T., van Strijp J.A.G. (2020). Studying Staphylococcal Leukocidins: A Challenging Endeavor. Front. Microbiol..

[16] Medzhitov R., Schneider D.S., Soares M.P. (2012). Disease Tolerance as a Defense Strategy. Science.

[17] Cortes-Bratti X., Frisan T., Thelestam M. (2001). The cytolethal distending toxins induce DNA damage and cell cycle arrest. Toxicon Off. J. Int. Soc. Toxinology.

[18] Jinadasa R.N., Bloom S.E., Weiss R.S., Duhamel G.E. (2011). Cytolethal distending toxin: A conserved bacterial genotoxin that blocks cell cycle progression, leading to apoptosis of a broad range of mammalian cell lineages. Microbiology.

[19] Nesic D., Hsu Y., Stebbins C.E. (2004). Assembly and function of a bacterial genotoxin. Nature.

[20] Elwell C.A., Dreyfus L.A. (2000). DNAase I homologous residues in CdtB are critical for cytolethal distending toxin-mediated cell cycle arrest. Mol. Microbiol..

[21] Lara-Tejero M., Galan J.E. (2000). A bacterial toxin that controls cell cycle progression as a deoxyribonuclease I-like protein. Science.

[22] Frisan T., Cortes-Bratti X., Chaves-Olarte E., Stenerlöw B., Thelestam M. (2003). The *Haemophilus ducreyi* cytolethal distending toxin induces DNA double strand breaks and promotes ATM-dependent activation of RhoA. Cell. Microbiol..

[23] Fowler C.C., Chang S.J., Gao X., Geiger T., Stack G., Galan J.E. (2017). Emerging insights into the biology of typhoid toxin. Curr. Opin. Microbiol..

[24] Fais T., Delmas J., Barnich N., Bonnet R., Dalmasso G. (2018). Colibactin: More Than a New Bacterial Toxin. Toxins.

[25] Guerra L., Teter K., Lilley B.N., Stenerlow B., Holmes R.K., Ploegh H.L., Sandvig K., Thelestam M., Frisan T. (2005). Cellular internalization of cytolethal distending toxin: A new end to a known pathway. Cell Microbiol..

[26] Boesze-Battaglia K., Besack D., McKay T., Zekavat A., Otis L., Jordan-Sciutto K., Shenker B.J. (2006). Cholesterol-rich membrane microdomains mediate cell cycle arrest induced by Actinobacillus actinomycetemcomitans cytolethal-distending toxin. Cell Microbiol..

[27] Lin C.D., Lai C.K., Lin Y.H., Hsieh J.T., Sing Y.T., Chang Y.C., Chen K.C., Wang W.C., Su H.L., Lai C.H. (2011). Cholesterol Depletion Reduces Entry of Campylobacter jejuni Cytolethal Distending Toxin and Attenuates Intoxication of Host Cells. Infect. Immun..

[28] McSweeney L.A., Dreyfus L.A. (2005). Carbohydrate-Binding specificity of the Escherichia coli cytolethal distending toxin CdtA-II and CdtC-II subunits. Infect. Immun..

[29] Frisan T. (2015). Bacterial genotoxins: The long journey to the nucleus of mammalian cells. Biochim. Biophys. Acta.

[30] Gargi A., Tamilselvam B., Powers B., Prouty M.G., Lincecum T., Eshraghi A., Maldonado-Arocho F.J., Wilson B.A., Bradley K.A., Blanke S.R. (2013). Cellular interactions of the cytolethal distending toxins from Escherichia coli and Haemophilus ducreyi. J. Biol. Chem..

[31] Boesze-Battaglia K., Dhingra A., Walker L.M., Zekavat A., Shenker B.J. (2020). Internalization and Intoxication of Human Macrophages by the Active Subunit of the Aggregatibacter actinomycetemcomitans Cytolethal Distending Toxin Is Dependent Upon Cellugyrin (Synaptogyrin-2). Front. Immunol..

[32] Chen M.X., Chen Y., Fu R., Mao G.Q., Liu S.Y., Shen T.B. (2020). Rab5a Promotes Cytolethal Distending Toxin B Induced Cytotoxicity and Inflammation. Infect. Immun..

[33] Eshraghi A., Dixon S.D., Tamilselvam B., Kim E.J., Gargi A., Kulik J.C., Damoiseaux R., Blanke S.R., Bradley K.A. (2014). Cytolethal distending toxins require components of the ER-associated degradation pathway for host cell entry. PLoS Pathog..

[34] Spano S., Ugalde J.E., Galan J.E. (2008). Delivery of a Salmonella Typhi exotoxin from a host intracellular compartment. Cell Host Microbe.

[35] Song J., Gao X., Galan J.E. (2013). Structure and function of the Salmonella Typhi chimaeric A(2)B(5) typhoid toxin. Nature.

[36] Desai P.T., Porwollik S., Long F., Cheng P., Wollam A., Clifton S.W., Weinstock G.M., McClelland M. (2013). Evolutionary Genomics of Salmonella enterica Subspecies. mBio.

[37] Suez J., Porwollik S., Dagan A., Marzel A., Schorr Y.I., Desai P.T., Agmon V., McClelland M., Rahav G., Gal-Mor O. (2013). Virulence Gene Profiling and Pathogenicity Characterization of Non-Typhoidal Salmonella Accounted for Invasive Disease in Humans. PLoS ONE.

[38] Rodriguez-Rivera L.D., Bowen B.M., den Bakker H.C., Duhamel G.E., Wiedmann M. (2015). Characterization of the cytolethal distending toxin (typhoid toxin) in non-typhoidal Salmonella serovars. Gut Pathog..

[39] Deng L.Q., Song J.M., Gao X., Wang J.W., Yu H., Chen X., Varki N., Naito-Matsui Y., Galan J.E., Varki A. (2014). Host Adaptation of a Bacterial Toxin from the Human Pathogen Salmonella Typhi. Cell.

[40] Chang S.J., Jin S.C., Jiao X.Y., Galan J.E. (2019). Unique features in the intracellular transport of typhoid toxin revealed by a genome-wide screen. PLoS Pathog..

[41] Guidi R., Levi L., Rouf S.F., Puiac S., Rhen M., Frisan T. (2013). *Salmonella enterica* delivers its genotoxin through outer membrane vesicles secreted from infected cells. Cell. Microbiol..

[42] Suresh A., Shaik S., Baddam R., Ranjan A., Qumar S., Jadhav S., Semmler T., Ghazi I.A., Wieler L.H., Ahmed N. (2021). Evolutionary Dynamics Based on Comparative Genomics of Pathogenic Escherichia coli Lineages Harboring Polyketide Synthase (pks) Island. mBio.

[43] Nougayrede J.P., Homburg S., Taieb F., Boury M., Brzuszkiewicz E., Gottschalk G., Buchrieser C., Hacker J., Dobrindt U., Oswald E. (2006). *Escherichia coli* induces DNA double-strand breaks in eukaryotic cells. Science.

[44] Xue M.Z., Kim C.S., Healy A.R., Wernke K.M., Wang Z.X., Frischling M.C., Shine E.E., Wang W.W., Herzon S.B., Crawford J.M. (2019). Structure elucidation of colibactin and its DNA cross-links. Science.

[45] Bossuet-Greif N., Vignard J., Taieb F., Mirey G., Dubois D., Petit C., Oswald E., Nougayrede J.P. (2018). The Colibactin Genotoxin Generates DNA Interstrand Cross-Links in Infected Cells. mBio.

[46] Healy A.R., Nikolayevskiy H., Patel J.R., Crawford J.M., Herzon S.B. (2016). A Mechanistic Model for Colibactin-Induced Genotoxicity. J. Am. Chem. Soc..

[47] Tripathi P., Shine E.E., Healy A.R., Kim C.S., Herzon S.B., Bruner S.D., Crawford J.M. (2017). ClbS Is a Cyclopropane Hydrolase That Confers Colibactin Resistance. J. Am. Chem. Soc..

[48] Reuter C., Alzheimer M., Walles H., Oelschlaeger T.A. (2018). An adherent mucus layer attenuates the genotoxic effect of colibactin. Cell. Microbiol..

[49] Blackford A.N., Jackson S.P. (2017). ATM, ATR, and DNA-PK: The Trinity at the Heart of the DNA Damage Response. Mol. Cell.

[50] Sancar A., Lindsey-Boltz L.A., Unsal-Kacmaz K., Linn S. (2004). Molecular mechanisms of mammalian DNA repair and the DNA damage checkpoints. Annu. Rev. Biochem..

[51] Walden H., Deans A.J. (2014). The Fanconi anemia DNA repair pathway: Structural and functional insights into a complex disorder. Annu. Rev. Biophys..

[52] Kitagawa T., Hoshida H., Akada R. (2007). Genome-Wide analysis of cellular response to bacterial genotoxin CdtB in yeast. Infect. Immun..

[53] Fedor Y., Vignard J., Nicolau-Travers M.L., Boutet-Robinet E., Watrin C., Salles B., Mirey G. (2013). From single-strand breaks to double-strand breaks during S-phase: A new mode of action of the Escherichia coli Cytolethal Distending Toxin. Cell. Microbiol..

[54] Fahrer J., Huelsenbeck J., Jaurich H., Dorsam B., Frisan T., Eich M., Roos W.P., Kaina B., Fritz G. (2014). Cytolethal distending toxin (CDT) is a radiomimetic agent and induces persistent levels of DNA double-strand breaks in human fibroblasts. DNA Repair.

[55] Pucci B., Kasten M., Giordano A. (2000). Cell cycle and apoptosis. Neoplasia.

[56] Green D.R., Llambi F. (2015). Cell Death Signaling. CSH Perspect. Biol..

[57] Shenker B.J., McKay T., Datar S., Miller M., Chowhan R., Demuth D. (1999). *Actinobacillus actinomycetemcomitans* immunosuppressive protein is a member of the family of cytolethal distending toxins capable of causing a G2 arrest in human T cells. J. Immunol..

[58] Shenker B.J., Hoffmaster R.H., Zekavat A., Yamaguchi N., Lally E.T., Demuth D.R. (2001). Induction of apoptosis in human T cells by *Actinobacillus actinomycetemcomitans* cytolethal distending toxin is a consequence of G2 arrest of the cell cycle. J. Immunol..

[59] Ohara M., Hayashi T., Kusunoki Y., Miyauchi M., Takata T., Sugai M. (2004). Caspase-2 and caspase-7 are involved in cytolethal distending toxin-induced apoptosis in Jurkat and MOLT-4 T-cell lines. Infect. Immun..

[60] Ohara M., Hayashi T., Kusunoki Y., Nakachi K., Fujiwara T., Komatsuzawa H., Sugai M. (2008). Cytolethal distending toxin induces caspase-dependent and -independent cell death in MOLT-4 cells. Infect. Immun..

[61] Svensson L., Tarkowski A., Thelestam M., Lagergård T. (2001). The impact of *Haemophilus ducreyi* cytolethal distending toxin on cells involved in immune response. Microb. Pathog..

[62] Wising C., Azem J., Zetterberg M., Svensson L.A., Ahlman K., Lagergard T. (2005). Induction of apoptosis/necrosis in various human cell lineages by Haemophilus ducreyi cytolethal distending toxin. Toxicon Off. J. Int. Soc. Toxinol..

[63] Li G., Niu H., Zhang Y.H., Li Y.L., Xie F., Langford P.R., Liu S., Wang C.L. (2017). Haemophilus parasuis cytolethal distending toxin induces cell cycle arrest and p53-dependent apoptosis. PLoS ONE.

[64] Shenker B.J., Boesze-Battaglia K., Scuron M.D., Walker L.P., Zekavat A., Dlakic M. (2016). The toxicity of the Aggregatibacter actinomycetemcomitans cytolethal distending toxin correlates with its phosphatidylinositol-3,4,5-triphosphate phosphatase activity. Cell. Microbiol..

[65] Shenker B.J., Walker L.M., Zekavat A., Weiss R.H., Boesze-Battaglia K. (2020). The Cell-Cycle Regulatory Protein p21(CIP1/WAF1) Is Required for Cytolethal Distending Toxin (Cdt)-Induced Apoptosis. Pathogens.

[66] Liyanage N.P.M., Manthey K.C., Dassanayake R.P., Kuszynski C.A., Oakley G.G., Duhamel G.E. (2010). Helicobacter hepaticus Cytolethal Distending Toxin Causes Cell Death in Intestinal Epithelial Cells via Mitochondrial Apoptotic Pathway. Helicobacter.

[67] Humphreys D., ElGhazaly M., Frisan T. (2020). Senescence and Host–Pathogen Interactions. Cells.

[68] Blazkova H., Krejcikova K., Moudry P., Frisan T., Hodny Z., Bartek J. (2010). Bacterial Intoxication Evokes Cellular Senescence with Persistent DNA Damage and Cytokine Signaling. J. Cell Mol. Med..

[69] Ibler A.E.M., Elghazaly M., Naylor K.L., Bulgakova N.A., El-Khamisy S.F., Humphreys D. (2019). Typhoid toxin exhausts the RPA response to DNA replication stress driving senescence and Salmonella infection. Nat. Commun..

[70] Secher T., Samba-Louaka A., Oswald E., Nougayrede J.P. (2013). *Escherichia coli* producing colibactin triggers premature and transmissible senescence in mammalian cells. PLoS ONE.

[71] Cougnoux A., Dalmasso G., Martinez R., Buc E., Delmas J., Gibold L., Sauvanet P., Darcha C., Dechelotte P., Bonnet M. (2014). Bacterial genotoxin colibactin promotes colon tumour growth by inducing a senescence-associated secretory phenotype. Gut.

[72] Dalmasso G., Cougnoux A., Delmas J., Darfeuille-Michaud A., Bonnet R. (2014). The bacterial genotoxin colibactin promotes colon tumor growth by modifying the tumor microenvironment. Gut Microbes.

[73] Pere-Vedrenne C., Prochazkova-Carlotti M., Rousseau B., He W., Chambonnier L., Sifre E., Buissonniere A., Dubus P., Megraud F., Varon C. (2017). The Cytolethal Distending Toxin Subunit CdtB of Helicobacter hepaticus Promotes Senescence and Endoreplication in Xenograft Mouse Models of Hepatic and Intestinal Cell Lines. Front. Cell. Infect. Microbiol..

[74] Hanahan D., Weinberg R.A. (2011). Hallmarks of cancer: The next generation. Cell.

[75] Guidi R., Guerra L., Levi L., Stenerlow B., Fox J.G., Josenhans C., Masucci M.G., Frisan T. (2013). Chronic exposure to the cytolethal distending toxins of Gram-negative bacteria promotes genomic instability and altered DNA damage response. Cell. Microbiol..

[76] Cuevas-Ramos G., Petit C.R., Marcq I., Boury M., Oswald E., Nougayrede J.P. (2010). Escherichia coli induces DNA damage in vivo and triggers genomic instability in mammalian cells. Proc. Natl. Acad. Sci. USA.

[77] Zhan T., Rindtorff N., Boutros M. (2017). Wnt signaling in cancer. Oncogene.

[78] Iftekhar A., Berger H., Bouznad N., Heuberger J., Boccellato F., Dobrindt U., Hermeking H., Sigal M., Meyer T.F. (2021). Genomic aberrations after short-term exposure to colibactin-producing *E. coli* transform primary colon epithelial cells. Nat. Commun..

[79] Muzny D.M., Bainbridge M., Chang K., Dinh H.H., Drummond J.A., Fowler G., Kovar C.L., Lewis L.R., Morgan M.B., Newsham I. (2012). Comprehensive molecular characterization of human colon and rectal cancer. Nature.

[80] Armaghany T., Wilson J.D., Chu Q., Mills G. (2012). Genetic alterations in colorectal cancer. Gastrointest. Cancer Res..

[81] Kiesler P., Fuss I.J., Strober W. (2015). Experimental Models of Inflammatory Bowel Diseases. Cell. Mol. Gastroenterol. Hepatol..

[82] Shen Z., Feng Y., Rogers A.B., Rickman B., Whary M.T., Xu S., Clapp K.M., Boutin S.R., Fox J.G. (2009). Cytolethal distending toxin promotes Helicobacter cinaedi-associated typhlocolitis in interleukin-10-deficient mice. Infect. Immun..

[83] Ge Z., Feng Y., Ge L., Parry N., Muthupalani S., Fox J.G. (2017). Helicobacter hepaticus cytolethal distending toxin promotes intestinal carcinogenesis in 129Rag2-deficient mice. Cell. Microbiol..

[84] Ge Z., Feng Y., Whary M.T., Nambiar P.R., Xu S., Ng V., Taylor N.S., Fox J.G. (2005). Cytolethal distending toxin is essential for Helicobacter hepaticus colonization in outbred Swiss Webster mice. Infect. Immun..

[85] Arthur J.C., Perez-Chanona E., Mühlbauer M., Tomkovich S., Uronis J.M., Fan T., Campbell B.J., Abujamel T., Dogan B., Rogers A.B. (2012). Intestinal inflammation targets cancer-inducing activity of the microbiota. Science.

[86] Arthur J.C., Gharaibeh R.Z., Muhlbauer M., Perez-Chanona E., Uronis J.M., McCafferty J., Fodor A.A., Jobin C. (2014). Microbial genomic analysis reveals the essential role of inflammation in bacteria-induced colorectal cancer. Nat. Commun..

[87] Dejea C.M., Wick E.C., Hechenbleikner E.M., White J.R., Welch J.L.M., Rossetti B.J., Peterson S.N., Snesrud E.C., Borisy G.G., Lazarev M. (2014). Microbiota organization is a distinct feature of proximal colorectal cancers. Proc. Natl. Acad. Sci. USA.

[88] Drewes J.L., White J.R., Dejea C.M., Fathi P., Iyadorai T., Vadivelu J., Roslani A.C., Wick E.C., Mongodin E.F., Loke M.F. (2017). High-Resolution bacterial 16S rRNA gene profile meta-analysis and biofilm status reveal common colorectal cancer consortia. NPJ Biofilms Microbiomes.

[89] Dejea C.M., Fathi P., Craig J.M., Boleij A., Taddese R., Geis A.L., Wu X., Shields C.E.D., Hechenbleikner E.M., Huso D.L. (2018). Patients with familial adenomatous polyposis harbor colonic biofilms containing tumorigenic bacteria. Science.

[90] Eklof V., Lofgren-Burstrom A., Zingmark C., Edin S., Larsson P., Karling P., Alexeyev O., Rutegard J., Wikberg M.L., Palmqvist R. (2017). Cancer-Associated fecal microbial markers in colorectal cancer detection. Int. J. Cancer.

[91] Warren R.L., Freeman D.J., Pleasance S., Watson P., Moore R.A., Cochrane K., Allen-Vercoe E., Holt R.A. (2013). Co-occurrence of anaerobic bacteria in colorectal carcinomas. Microbiome.

[92] Allali I., Delgado S., Marron P.I., Astudillo A., Yeh J.J., Ghazal H., Amzazi S., Keku T., Azcarate-Peril M.A. (2015). Gut microbiome compositional and functional differences between tumor and non-tumor adjacent tissues from cohorts from the US and Spain. Gut Microbes.

[93] He Z., Gharaibeh R., Newsome R., Pope J., Dougherty M., Tomkovich S., Pons B., Mirey G.J.V., Hendrixson D., Vignard J. (2018). Campylobacterjejuni promotes colorectal tumorigenesis through the action of cytolethal distending toxin. Gut.

[94] Pere-Vedrenne C., Cardinaud B., Varon C., Mocan I., Buissonniere A., Izotte J., Megraud F., Menard A. (2016). The Cytolethal Distending Toxin Subunit CdtB of Helicobacter Induces a Th17-related and Antimicrobial Signature in Intestinal and Hepatic Cells In Vitro. J. Infect. Dis..

[95] Akifusa S., Poole S., Lewthwaite J., Henderson B., Nair S.P. (2001). Recombinant Actinobacillus actinomycetemcomitans cytolethal distending toxin proteins are required to interact to inhibit human cell cycle progression and to stimulate human leukocyte cytokine synthesis. Infect. Immun..

[96] Shenker B.J., Ojcius D.M., Walker L.P., Zekavat A., Scuron M.D., Boesze-Battaglia K. (2015). Aggregatibacter actinomycetemcomitans cytolethal distending toxin activates the NLRP3 inflammasome in human macrophages, leading to the release of proinflammatory cytokines. Infect. Immun..

[97] Ando-Suguimoto E.S., da Silva M.P., Kawamoto D., Chen C., DiRienzo J.M., Mayer M.P. (2014). The cytolethal distending toxin of Aggregatibacter actinomycetemcomitans inhibits macrophage phagocytosis and subverts cytokine production. Cytokine.

[98] Belibasakis G.N., Johansson A., Wang Y., Chen C., Lagergard T., Kalfas S., Lerner U.H. (2005). Cytokine responses of human gingival fibroblasts to Actinobacillus actinomycetemcomitans cytolethal distending toxin. Cytokine.

[99] Hickey T.E., McVeigh A.L., Scott D.A., Michielutti R.E., Bixby A., Carroll S.A., Bourgeois A.L., Guerry P. (2000). Campylobacter jejuni cytolethal distending toxin mediates release of interleukin-8 from intestinal epithelial cells. Infect. Immun..

[100] Zheng J., Meng J., Zhao S., Singh R., Song W. (2008). Campylobacter-induced interleukin-8 secretion in polarized human intestinal epithelial cells requires Campylobacter-secreted cytolethal distending toxin- and Toll-like receptor-mediated activation of NF-kappaB. Infect. Immun..

[101] Fox J.G., Rogers A.B., Whary M.T., Ge Z., Taylor N.S., Xu S., Horwitz B.H., Erdman S.E. (2004). Gastroenteritis in NF-kappaB-deficient mice is produced with wild-type Camplyobacter jejuni but not with *C. jejuni* lacking cytolethal distending toxin despite persistent colonization with both strains. Infect. Immun..

[102] Pratt J.S., Sachen K.L., Wood H.D., Eaton K.A., Young V.B. (2006). Modulation of host immune responses by the cytolethal distending toxin of Helicobacter hepaticus. Infect. Immun..

[103] Young V.B., Knox K.A., Pratt J.S., Cortez J.S., Mansfield L.S., Rogers A.B., Fox J.G., Schauer D.B. (2004). In vitro and in vivo characterization of Helicobacter hepaticus cytolethal distending toxin mutants. Infect. Immun..

[104] Ge Z., Rogers A.B., Feng Y., Lee A., Xu S., Taylor N.S., Fox J.G. (2007). Bacterial cytolethal distending toxin promotes the development of dysplasia in a model of microbially induced hepatocarcinogenesis. Cell. Microbiol..

[105] Secher T., Payros D., Brehin C., Boury M., Watrin C., Gillet M., Bernard-Cadenat I., Menard S., Theodorou V., Saoudi A. (2015). Oral tolerance failure upon neonatal gut colonization with Escherichia coli producing the genotoxin colibactin. Infect. Immun..

[106] Bakthavatchalu V., Wert K.J., Feng Y., Mannion A., Ge Z.M., Garcia A., Scott K.E., Caron T.J., Madden C.M., Jacobsen J.T. (2018). Cytotoxic *Escherichia coli* strains encoding colibactin isolated from immunocompromised mice with urosepsis and meningitis. PLoS ONE.

[107] Guerra L., Carr H.S., Richter-Dahlfors A., Masucci M.G., Thelestam M., Frost J.A., Frisan T. (2008). A bacterial cytotoxin identifies the RhoA exchange factor Net1 as a key effector in the response to DNA damage. PLoS ONE.

[108] Tak P.P., Firestein G.S. (2001). NF-kappa B: A key role in inflammatory diseases. J. Clin. Investig..

[109] Cuenda A., Rousseau S. (2007). p38 MAP-kinases pathway regulation, function and role in human diseases. Biochim. Biophys. Acta.

[110] Yu H., Pardoll D., Jove R. (2009). STATs in cancer inflammation and immunity: A leading role for STAT3. Nat. Rev. Cancer.

[111] Ahn J., Gutman D., Saijo S., Barber G.N. (2012). STING manifests self DNA-dependent inflammatory disease. Proc. Natl. Acad. Sci. USA.

[112] Lan Y.Y., Londono D., Bouley R., Rooney M.S., Hacohen N. (2014). Dnase2a deficiency uncovers lysosomal clearance of damaged nuclear DNA via autophagy. Cell Rep..

[113] Hartlova A., Erttmann S.F., Raffi F.A., Schmalz A.M., Resch U., Anugula S., Lienenklaus S., Nilsson L.M., Kroger A., Nilsson J.A. (2015). DNA damage primes the type I interferon system via the cytosolic DNA sensor STING to promote anti-microbial innate immunity. Immunity.

[114] Harding S.M., Benci J.L., Irianto J., Discher D.E., Minn A.J., Greenberg R.A. (2017). Mitotic progression following DNA damage enables pattern recognition within micronuclei. Nature.

[115] Morrison P.J., Bending D., Fouser L.A., Wright J.F., Stockinger B., Cooke A., Kullberg M.C. (2013). Th17-cell plasticity in Helicobacter hepaticus-induced intestinal inflammation. Mucosal Immunol..

[116] Eberl G. (2016). Immunity by equilibrium. Nat. Rev. Immunol..

[117] Annunziato F., Romagnani C., Romagnani S. (2015). The 3 major types of innate and adaptive cell-mediated effector immunity. J. Allergy Clin. Immunol..

[118] Gelfanova V., Hansen E.J., Spinola S.M. (1999). Cytolethal distending toxin of *Haemophilus ducreyi* induces apoptotic death of Jurkat T cells. Infect. Immun..

[119] Marcq I., Martin P., Payros D., Cuevas-Ramos G., Boury M., Watrin C., Nougayrede J.P., Olier M., Oswald E. (2014). The Genotoxin Colibactin Exacerbates Lymphopenia and Decreases Survival Rate in Mice Infected With Septicemic *Escherichia coli*. J. Infect. Dis..

[120] Johnson J.R., Johnston B., Kuskowski M.A., Nougayrede J.P., Oswald E. (2008). Molecular Epidemiology and Phylogenetic Distribution of the Escherichia coli pks Genomic Island. J. Clin. Microbiol..

[121] Olier M., Marcq I., Salvador-Cartier C., Secher T., Dobrindt U., Boury M., Bacquie V., Penary M., Gaultier E., Nougayrede J.P. (2012). Genotoxicity of Escherichia coli Nissle 1917 strain cannot be dissociated from its probiotic activity. Gut Microbes.

[122] Del Bel Belluz L., Guidi R., Pateras I.S., Levi L., Mihaljevic B., Rouf S.F., Wrande M., Candela M., Turroni S., Nastasi C. (2016). The Typhoid Toxin Promotes Host Survival and the Establishment of a Persistent Asymptomatic Infection. PLoS Pathog..

[123] Miller R.A., Betteken M.I., Guo X., Altier C., Duhamel G.E., Wiedmann M. (2018). The Typhoid Toxin Produced by the Nontyphoidal Salmonella enterica Serotype Javiana Is Required for Induction of a DNA Damage Response In Vitro and Systemic Spread In vivo. mBio.

[124] Martin O.C., Bergonzini A., Chiloeches M.L., Paparouna E., Butter D., Theodorou S.D., Haykal M.M., Boutet-Robinet E., Tebaldi T., Wakeham A. (2021). Influence of the microenvironment on modulation of the host response by typhoid toxin. Cell Rep..

[125] Ostolaza H., Gonzalez-Bullon D., Uribe K.B., Martin C., Amuategi J., Fernandez-Martinez X. (2019). Membrane Permeabilization by Pore-Forming RTX Toxins: What Kind of Lesions Do These Toxins Form?. Toxins.

[126] Dal Peraro M., van der Goot F.G. (2016). Pore-forming toxins: Ancient, but never really out of fashion. Nat. Rev. Microbiol..

[127] Broz P., Pelegrin P., Shao F. (2020). The gasdermins, a protein family executing cell death and inflammation. Nat. Rev. Immunol..

[128] Hachim M.Y., Khalil B.A., Elemam N.M., Maghazachi A.A. (2020). Pyroptosis: The missing puzzle among innate and adaptive immunity crosstalk. J. Leukoc. Biol..

[129] von Hoven G., Rivas A.J., Neukirch C., Meyenburg M., Qin Q.Q., Parekh S., Hellmann N., Husmann M. (2017). Repair of a Bacterial Small beta-Barrel Toxin Pore Depends on Channel Width. mBio.

[130] Wai S.N., Lindmark B., Soderblom T., Takade A., Westermark M., Oscarsson J., Jass J., Richter-Dahlfors A., Mizunoe Y., Uhlin B.E. (2003). Vesicle-Mediated export and assembly of pore-forming oligomers of the enterobacterial ClyA cytotoxin. Cell.

[131] Uhlen P., Laestadius A., Jahnukainen T., Soderblom T., Backhed F., Celsi G., Brismar H., Normark S., Aperia A., Richter-Dahlfors A. (2000). Alpha-Haemolysin of uropathogenic E-coli induces Ca2+ oscillations in renal epithelial cells. Nature.

[132] Soderblom T., Oxhamre C., Wai S.N., Uhlen P., Aperia A., Uhlin B.E., Richter-Dahlfors A. (2005). Effects of the Escherichia coli toxin cytolysin A on mucosal immunostimulation via epithelial Ca2+ signalling and Toll-like receptor 4. Cell. Microbiol..

[133] Ristow L.C., Welch R.A. (2016). Hemolysin of uropathogenic Escherichia coli: A cloak or a dagger?. BBA Biomembr..

[134] Oscarsson J., Mizunoe Y., Uhlin B.E., Haydon D.J. (1996). Induction of haemolytic activity in Escherichia coli by the slyA gene product. Mol. Microbiol..

[135] Hamon M.A., Ribet D., Stavru F., Cossart P. (2012). Listeriolysin O: The Swiss army knife of Listeria. Trends Microbiol..

[136] Repp H., Pamukci Z., Koschinski A., Domann E., Darji A., Birringer J., Brockmeier D., Chakraborty T., Dreyer F. (2002). Listeriolysin of Listeria monocytogenes forms Ca2+-permeable pores leading to intracellular Ca2+ oscillations. Cell. Microbiol..

[137] Gekara N.O., Westphal K., Ma B., Rohde M., Groebe L., Weiss S. (2007). The multiple mechanisms of Ca2+ signalling by listeriolysin O, the cholesterol-dependent cytolysin of Listeria monocytogenes. Cell. Microbiol..

[138] Krause K.H., Fivaz M., Monod A., van der Goot F.G. (1998). Aerolysin induces G-protein activation and Ca2+ release from intracellular stores in human granulocytes. J. Biol. Chem..

[139] Wiles T.J., Mulvey M.A. (2013). The RTX pore-forming toxin alpha-hemolysin of uropathogenic *Escherichia coli*: Progress and perspectives. Future Microbiol..

[140] Tang P., Rosenshine I., Cossart P., Finlay B.B. (1996). Listeriolysin O activates mitogen-activated protein kinase in eucaryotic cells. Infect. Immun..

[141] Huffman D.L., Abrami L., Sasik R., Corbeil J., van der Goot F.G., Aroian R.V. (2004). Mitogen-Activated protein kinase pathways defends against bacterial pore-forming toxins. Proc. Natl. Acad. Sci. USA.

[142] Ratner A.J., Hippe K.R., Aguilar J.L., Bender M.H., Nelson A.L., Weiser J.N. (2006). Epithelial cells are sensitive detectors of bacterial pore-forming toxins. J. Biol. Chem..

[143] Gonzalez M.R., Bischofberger M., Freche B., Ho S., Parton R.G., van der Goot F.G. (2011). Pore-Forming toxins induce multiple cellular responses promoting survival. Cell. Microbiol..

[144] Kao C.Y., Los F.C.O., Huffman D.L., Wachi S., Kloft N., Husmann M., Karabrahimi V., Schwartz J., Bellier A., Ha C. (2011). Global Functional Analyses of Cellular Responses to Pore-Forming Toxins. PLoS Pathog..

[145] Cabezas S., Ho S., Ros U., Lanio M.E., Alvarez C., van der Goot F.G. (2017). Damage of eukaryotic cells by the pore-forming toxin sticholysin II: Consequences of the potassium efflux. BBA Biomembr..

[146] Husmann M., Dersch K., Bobkiewicz W., Beckmann E., Veerachato G., Bhakdi S. (2006). Differential role of p38 mitogen activated protein kinase for cellular recovery from attack by pore-forming S-aureus alpha-toxin or streptolysin O. Biochem. Biophys. Res. Commun..

[147] Wiles T.J., Dhakal B.K., Eto D.S., Mulvey M.A. (2008). Inactivation of host Akt/protein kinase B signaling by bacterial pore-forming toxins. Mol. Biol. Cell.

[148] Hers I., Vincent E.E., Tavare J.M. (2011). Akt signalling in health and disease. Cell. Signal..

[149] Vergadi E., Ieronymaki E., Lyroni K., Vaporidi K., Tsatsanis C. (2017). Akt Signaling Pathway in Macrophage Activation and M1/M2 Polarization. J. Immunol..

[150] Rajaee A., Barnett R., Cheadle W.G. (2018). Pathogen- and Danger-Associated Molecular Patterns and the Cytokine Response in Sepsis. Surg. Infect..

[151] Konig B., Konig W. (1993). Induction and Suppression of Cytokine Release (Tumor-Necrosis-Factor-Alpha—Interleukin-6, Interleukin-1-Beta) by *Escherichia-coli* Pathogenicity Factors (Adhesions, Alpha-Hemolysin). Immunology.

[152] Bhushan S., Hossain H., Lu Y.N., Geisler A., Tchatalbachev S., Mikulski Z., Schuler G., Klug J., Pilatz A., Wagenlehner F. (2011). Uropathogenic *E. coli* Induce Different Immune Response in Testicular and Peritoneal Macrophages: Implications for Testicular Immune Privilege. PLoS ONE.

[153] Walker J.A., McKenzie A.N.J. (2018). TH2 cell development and function. Nat. Rev. Immunol..

[154] Bebien M., Hensler M.E., Davanture S., Hsu L.C., Karin M., Park J.M., Alexopoulou L., Liu G.Y., Nizet V., Lawrence T. (2012). The pore-forming toxin beta hemolysin/cytolysin triggers p38 MAPK-dependent IL-10 production in macrophages and inhibits innate immunity. PLoS Pathog..

[155] Hancz D., Westerlund E., Valfridsson C., Aemero G.M., Bastiat-Sempe B., Orning P., Lien E., Wessels M.R., Persson J.J. (2019). Streptolysin O Induces the Ubiquitination and Degradation of Pro-IL-1beta. J. Innate Immun..

[156] Bhattacharjee P., Keyel P.A. (2018). Cholesterol-Dependent cytolysins impair pro-inflammatory macrophage responses. Sci. Rep..

[157] Kroczek R.A., Mages H.W., Hutloff A. (2004). Emerging paradigms of T-cell co-stimulation. Curr. Opin. Immunol..

[158] Woida P.J., Satchell K.J.F. (2020). The Vibrio cholerae MARTX toxin silences the inflammatory response to cytoskeletal damage before inducing actin cytoskeleton collapse. Sci. Signal..

[159] Kim B.S., Gavin H.E., Satchell K.J.F. (2015). Distinct Roles of the Repeat-Containing Regions and Effector Domains of the Vibrio vulnificus Multifunctional-Autoprocessing Repeats-in-Toxin (MARTX) Toxin. mBio.

[160] Nishimoto A.T., Rosch J.W., Tuomanen E.I. (2020). Pneumolysin: Pathogenesis and Therapeutic Target. Front. Microbiol..

[161] Subramanian K., Neill D.R., Malak H.A., Spelmink L., Khandaker S., Marchiori G.D.L., Dearing E., Kirby A., Yang M., Achour A. (2019). Pneumolysin binds to the mannose receptor C type 1 (MRC-1) leading to anti-inflammatory responses and enhanced pneumococcal survival. Nat. Microbiol..

[162] Whibley N., Tucci A., Powrie F. (2019). Regulatory T cell adaptation in the intestine and skin. Nat. Immunol..

[163] Koffel R., Wolfmeier H., Larpin Y., Besancon H., Schoenauer R., Babiychuk V.S., Drucker P., Pabst T., Mitchell T.J., Babiychuk E.B. (2018). Host-Derived Microvesicles Carrying Bacterial Pore-Forming Toxins Deliver Signals to Macrophages: A Novel Mechanism of Shaping Immune Responses. Front. Immunol..

[164] Guermonprez P., Valladeau J., Zitvogel L., Thery C., Amigorena S. (2002). Antigen presentation and T cell stimulation by dendritic cells. Annu. Rev. Immunol..

[165] Djekic A., Muller A. (2016). The Immunomodulator VacA Promotes Immune Tolerance and Persistent Helicobacter pylori Infection through Its Activities on T-Cells and Antigen-Presenting Cells. Toxins.

[166] Oertli M., Noben M., Engler D.B., Semper R.P., Reuter S., Maxeiner J., Gerhard M., Taube C., Muller A. (2013). Helicobacter pylori gamma-glutamyl transpeptidase and vacuolating cytotoxin promote gastric persistence and immune tolerance. Proc. Natl. Acad. Sci. USA.

[167] Weiss G., Forster S., Irving A., Tate M., Ferrero R.L., Hertzog P., Frokiaer H., Kaparakis-Liaskos M. (2013). Helicobacter pylori VacA suppresses Lactobacillus acidophilus-induced interferon beta signaling in macrophages via alterations in the endocytic pathway. mBio.

[168] Molinari M., Salio M., Galli C., Norais N., Rappuoli R., Lanzavecchia A., Montecucco C. (1998). Selective inhibition of Ii-dependent antigen presentation by Helicobacter pylori toxin VacA. J. Exp. Med..

[169] Ross S.H., Cantrell D.A. (2018). Signaling and Function of Interleukin-2 in T Lymphocytes. Annu. Rev. Immunol..

[170] Gebert B., Fischer W., Weiss E., Hoffmann R., Haas R. (2003). *Helicobacter pylori* vacuolating cytotoxin inhibits T lymphocyte activation. Science.

[171] Brownlie R.J., Zamoyska R. (2013). T cell receptor signalling networks: Branched, diversified and bounded. Nat. Rev. Immunol..

[172] Gekara N.O., Zietara N., Geffers R., Weiss S. (2010). Listeria monocytogenes Induces T Cell Receptor Unresponsiveness through Pore-Forming Toxin Listeriolysin O. J. Infect. Dis..

[173] Gekara N.O., Groebe L., Viegas N., Weiss S. (2008). Listeria monocytogenes desensitizes immune cells to subsequent Ca2+ signaling via listeriolysin O-induced depletion of intracellular Ca2+ stores. Infect. Immun..

[174] Wolf A.I., Strauman M.C., Mozdzanowska K., Williams K.L., Osborne L.C., Shen H., Liu Q., Garlick D., Artis D., Hensley S.E. (2014). Pneumolysin expression by streptococcus pneumoniae protects colonized mice from influenza virus-induced disease. Virology.

[175] Laabei M., Uhlemann A.-C., Lowy F.D., Austin E.D., Yokoyama M., Ouadi K., Feil E., Thorpe H.A., Williams B., Perkins M. (2015). Evolutionary Trade-Offs Underlie the Multi-faceted Virulence of *Staphylococcus aureus*. PLoS Biol..

[176] Das S., Lindemann C., Young B.C., Muller J., Österreich B., Ternette N., Winkler A., Paprotka K., Reinhardt R., Förstner K.U. (2016). Natural mutations in a Staphylococcus aureus virulence regulator attenuate cytotoxicity but permit bacteremia and abscess formation. Proc. Natl. Acad. Sci. USA.

[177] Caiazza N.C., O’Toole G.A. (2003). Alpha-Toxin is required for biofilm formation by *Staphylococcus aureus*. J. Bacteriol..

[178] Szczesny P., Iacovache I., Muszewska A., Ginalski K., van der Goot F.G., Grynberg M. (2011). Extending the Aerolysin Family: From Bacteria to Vertebrates. PLoS ONE.

[179] Verster K.I., Wisecaver J.H., Karageorgi M., Duncan R.P., Gloss A.D., Armstrong E.E., Price D.K., Menon A.R., Ali Z.M., Whiteman N.K. (2019). Horizontal Transfer of Bacterial Cytolethal Distending Toxin B Genes to Insects. Mol. Biol. Evol..

[180] Oliver K.M., Degnan P.H., Burke G.R., Moran N.A. (2010). Facultative Symbionts in Aphids and the Horizontal Transfer of Ecologically Important Traits. Annu. Rev. Entomol..

[181] Gao Z.H., Deng C.J., Xie Y.Y., Guo X.L., Wang Q.Q., Liu L.Z., Lee W.H., Li S.A., Zhang Y. (2019). Pore-Forming toxin-like protein complex expressed by frog promotes tissue repair. FASEB J..

[182] Gieseck R.L., Wilson M.S., Wynn T.A. (2018). Type 2 immunity in tissue repair and fibrosis. Nat. Rev. Immunol..

